# ﻿Reinstatement of *Hellwigia* Warb. (Zingiberaceae) and its molecular and morphological delimitation

**DOI:** 10.3897/phytokeys.261.151948

**Published:** 2025-08-22

**Authors:** Seni Kurnia Senjaya, Axel Dalberg Poulsen, Marlina Ardiyani, Quentin C.B. Cronk

**Affiliations:** 1 Department of Botany, University of British Columbia, Vancouver, BC, V6T 1Z4, Canada; 2 Biodiversity Research Centre, University of British Columbia, Vancouver, BC, V6T 1Z4, Canada; 3 Research Center for Biosystematics and Evolution, Badan Riset dan Inovasi Nasional (BRIN), Bogor, West Java, 16911, Indonesia; 4 Royal Botanic Garden Edinburgh, Scotland, EH3 5LR, UK; 5 Beaty Biodiversity Museum, University of British Columbia, Vancouver, BC, V6T 1Z1, Canada

**Keywords:** *
Alpinia
*, *
Hellwigia
*, Huxley’s Line, ribosomal DNA, Zingiberaceae

## Abstract

The currently polyphyletic genus *Alpinia* Roxb. (Zingiberaceae) has over 260 species widely spread through subtropical and tropical Asia and a complex taxonomic history. This study focuses on the “Carolinensis” clade of *Alpinia* hitherto suggested by molecular evidence. We expand on previous research through more comprehensive taxon sampling (about a fivefold increase) and morphological observation. We inferred maximum likelihood (ML) and Bayesian trees based on the nuclear ribosomal internal transcribed spacer (ITS) region, using sequences available on GenBank along with 70 newly generated sequences from this study. Our main findings are as follows: (1) ITS provides a convenient marker for separating members of this clade from other *Alpinieae*. (2) Phylogenetic reconstruction strongly supports the monophyly of the Carolinensis clade. (3) We identify four subclades with relatively distinct geographical distributions. (4) We determine that morphologically the clade is characterized by inflorescences having densely packed flowers arranged on lateral monochasial cymes, tubular bracteoles, and flowers with oblong or linear labella. (5) The clade is apparently restricted to the east of Huxley’s biogeographic line. These findings support the recognition of the Carolinensis clade as a distinct genus. *Hellwigia* Warb. is the oldest valid name for this lineage, and till the present paper only includes *H. pulchra* Warb. We present a generic circumscription of the genus comprising 76 species to date placed in *Alpinia*. In the present paper, we make 76 new combinations and designate 26 lectotypes. The eastern distribution of the clade offers insights into historical biogeographic barriers and lineage diversification. As such, *Hellwigia* represents an interesting model for exploring evolutionary and ecological processes in this region, and future phylogenomic studies will be critical for resolving remaining phylogenetic uncertainties and for deepening our understanding of the evolutionary dynamics in this unique lineage.

## ﻿Introduction

*Alpinia* Roxb. (Zingiberaceae) is a genus comprising over 260 species predominantly found in tropical Asia (https://padme.rbge.org.uk/zrc/). The genus is placed within the subfamily Alpinioideae, which is characterized by leaves arranged flat and perpendicular to the rhizome and the reduced or absent lateral staminodes (Kress et al. 2002). Within this subfamily, *Alpinia* is part of the tribe Alpinieae, whose members are distinguished by fleshy indehiscent fruits and traditionally by the absence of extrafloral nectaries (Kress et al. 2002). Traditionally, the genus has been defined by its inflorescence emerging from the apex of a leafy shoot (Schumann 1904; Smith 1985), although exceptions exist in the radically flowering *Alpinia melichroa* K.Schum. and *A. pumila* Hook.f. (as well as occasionally in *A. japonica* (Thunb.) Miq.). *Alpinia* is also notable for its floral structures and diverse reproductive strategies, including flexistyly, monoecy, and andromonoecy, which have drawn significant botanical interest (Burtt and Smith 1972b; Sakai et al. 1999; Li et al. 2001).

The generic name of *Alpinia* was first used by Linnaeus (1753) for a single species from South America, *A. racemosa* L. However, over time, the name came to be applied primarily to species from Asia. As more species, mainly Asiatic, were added to *Alpinia*, American species were classified under the genus *Renealmia*. This shift was later formalized by Schumann (1904), who retained the name *Alpinia* for Asiatic species and placed *A. racemosa* as *Renealmia racemosa* (L.) A.Rich., thereby excluding the original type species from *Alpinia*. The name *Alpinia* was subsequently conserved for Asiatic species as *Alpinia* Roxb., with *Alpinia galanga* (L.) Willd. designated as the type (Burtt 1966, Burtt and Smith 1972a). Schumann (1904) classified *Alpinia* into five subgenera and 27 sections, using the morphology of the bract and bracteole as key diagnostic characters. In the same work, Schumann also synonymized several previously recognized genera in *Alpinia* (i.e., *Albina* Giseke, *Amomumsensu* C.Presl, *Buekia* Giseke, *Catimbium* Juss., *Cenolophon* Blume, *Galanga* Salisb., *Guillainia* Vieill., *Hellenia* Willd., *Hellwigia* Warb., *Heritiera* Retz., *Kolowratia* C.Presl, *Languas* J.Koenig, *Martensia* Giseke, and *Zerumbet* J.C.Wendl.).

Valeton (1913), in his study of New Guinea species, reclassified the subgenus Dieramalpinia, further subdivided *Eubractea* into subsections *Eustales* and *Kolowratia*, and introduced a new section, *Monanthocrater*. Subsequent efforts aimed to elevate morphologically distinct species groups within *Alpinia* to the generic rank. Notably, Ridley (1916) resurrected *Kolowratia* C.Presl and *Guillainia* Vieill. and introduced *Eriolopha* Ridl. as a new genus. Later, Holttum (1950) refined the classification by retaining species with funnel-shaped bracteoles in *Alpinia* and reassigning others to separate genera, including *Catimbium* Juss., *Cenolophon* Blume, and *Languas* J.Koenig. Despite these attempts, later taxonomists largely returned to Schumann’s broader concept of *Alpinia* until Rosemary M. Smith recognized the separation of *Pleuranthodium* (K.Schum.) R.M.Sm. from *Alpinia* and introduced a revised infrageneric classification (Smith 1990a, 1990b, 1991b). Smith (1990a) divided *Alpinia* into two subgenera based on the character of the labellum (petaloid or non-petaloid): the subgenus Alpinia, which consists of seven sections and ten subsections, and the subgenus Dieramalpinia, which includes four sections and two subsections. Smith’s classification remained the most widely accepted framework for *Alpinia* until further developments in molecular-based studies challenged this classification.

In the last 25 years, several studies have indicated that *Alpinia* and the related genus *Amomum* Roxb. are non-monophyletic (Rangsiruji et al. 2000, Kress et al. 2002, 2005). *Alpinia* comprises six clades within the Alpinioideae lineage, which have been informally named Galanga, Fax, Rafflesiana, Eubractea, Zerumbet, and Carolinensis (Kress et al. 2005, 2007). Discrepancies in morphological features and limited taxon sampling in those studies, however, hindered further taxonomic refinements (Kress et al. 2007). This finding prompted deeper investigations into various groups within the tribe Alpinieae. de Boer et al. (2018) studied *Amomum* s.l. and recircumscribed *Amomum* s.s., recognizing six genera (including three new genera). Docot et al. (2019b, 2019a) reinstated *Adelmeria* Ridl. and recircumscribed *Vanoverberghia* Merr. within the *Alpinia* “Eubractea” clade, and Poulsen et al. (2018, 2024) further investigated the *Alpinia* “Fax” clade, separating a new genus, *Sulettaria* A.D.Poulsen & Mathisen, and recircumscribing *Elettaria* Maton to include seven species, one of which is *A. fax* B.L.Burtt & R.M.Sm. As a result, *Alpinia* currently comprises five clades, one of which is the Carolinensis clade, the focus of the present paper.

Kress et al. (2005, 2007) originally identified the *Alpinia* “Carolinensis” clade using seven accessions, mainly distributed in Sulawesi. This clade is characterized by its large size, caducous primary inflorescence bracts, and a narrow, fleshy labellum pressed against the stamen, aligning morphologically with Alpinia section Myriocrater as defined by Smith (Kress et al. 2007). The species belonging to that section are found east of the Wallace’s Line (Wallace 1863), and most are believed to have a monoecious sexual system (Burtt and Smith 1972b; Smith 1977). Taxonomic refinement is needed for this clade, as with others, but limited sampling in previous studies has hindered such efforts and restricted evolutionary research, including studies of trait evolution. This clade is particularly interesting because it provides a unique opportunity to study the evolution of sexual systems within the ginger family and to explore how geographical factors drive diversification and speciation.

In this study, we aim to (1) expand the taxon sampling and test the monophyly of the Carolinensis clade; (2) characterize and identify species that belong to the clade using suitable molecular and morphological markers; (3) redetermine the position of the Carolinensis clade relative to other clades in Alpinioideae; (4) reveal any subclade structure within the Carolinensis clade, which may have bearings on future phylogenetic work; and (5) refine the taxonomic treatment of the clade.

## ﻿Materials and methods

### ﻿Plant materials

We extracted sequences and examined 70 accessions representing approximately 44 species (Suppl. material 4). We identified species using morphological features and molecular data, referring to type specimens, protologues, and relevant taxonomic literature. We sourced accessions from several botanic gardens, including Bogor Botanic Garden, Bali Botanic Garden, Royal Botanic Garden Edinburgh, and Fairchild Tropical Botanic Garden. Additionally, we collected samples during field explorations in eastern Indonesia, Papua New Guinea, and the Solomon Islands.

### ﻿DNA extraction and amplification

We extracted total DNA using the DNeasy Plant Mini Kit (Qiagen). Polymerase chain reaction (PCR) amplification of total DNA was performed with a total volume of 25 μl. Each mixture contains 12.5 μl KOD Hot Start Master Mix (Millipore), 1.25 μl of each primer (10 mM), 8 μl nuclease-free water, and 2 μl template DNA. The primer pairs used for amplification and sequencing were ITS4 and ITS5 (White et al. 1990). If these primers were ineffective, we utilized additional internal primers, ITS2P and ITS3P (Möller and Cronk 1997). We amplified the nuclear ribosomal Internal Transcribed Spacer (ITS) with the following PCR protocol: 95 °C 5 min, (95 °C 30 s, 50 °C 1 min, 72 °C 1 min) × 35, 72 °C 5 min. Sequencing was performed by Eurofins (Kentucky, USA).

### ﻿Phylogenetic analysis

We manually assembled contigs using Geneious Prime 2023.2 (https://www.geneious.com) and retrieved 231 sequences from alignments of three publications (de Boer et al. 2018; Poulsen et al. 2018; Docot et al. 2019b) available on Dryad (https://datadryad.org/stash). We performed sequence alignment using MAFFT v7.526 (Katoh et al. 2019) and partitioned the alignment into three regions: ITS1, 5.8S, and ITS2. We determined the most appropriate substitution models for each region using jModelTest (Posada 2008), with the Bayesian Information Criterion (BIC) applied for model selection. The best partition scheme was identified using the ModelFinder (Kalyaanamoorthy et al. 2017) implemented in IQ-TREE v2.3.6 (Minh et al. 2020). We reconstructed phylogenetic trees using maximum likelihood (ML) and Bayesian approaches. We performed the ML analysis in RAxML-NG (Kozlov et al. 2019). For the ITS1 and ITS2 regions, we applied the Tamura-Nei (TN93) substitution models with gamma distribution with four rate categories (G4). For the 5.8S region, the transition-transversion parameter 2 model (TPM2) was used with a proportion of invariant sites (I) and G4. We assessed branch support using 1,000 replicates with the standard nonparametric bootstrap (Felsenstein 1985) and a tree seed value of 12345.

We employed MrBayes v3.2.7 (Ronquist et al. 2012) for Bayesian phylogenetic analysis. We applied the symmetrical substitution model (SYM) combined with G4 for the ITS1 and ITS2 regions, while SYM with I and G4 was used for the 5.8S region. To ensure reproducibility, we specified random seeds (seed = 12345 and swapseed = 67890). The Markov chain Monte Carlo (MCMC) analysis was run for 5,000,000 generations, with two independent runs, each comprising four chains and a heating temperature of 0.02. Trees were sampled every 200 generations, and convergence was monitored using the standard deviation of split frequencies. We set the analysis to terminate when the split frequency fell below 0.01. We discarded the first 25% of samples as burn-in to remove initial parameter fluctuations. We summarized posterior parameter estimates and constructed a majority-rule consensus tree with a minimum partition frequency of 0.5 to resolve compatible clades.

We visualized the resulting phylogenetic trees from both analyses using FigTree (https://tree.bio.ed.ac.uk/software/figtree/). For enhanced visualization, we modified the trees using the *ggtree* package in R (Yu et al. 2017) and completed final adjustments and refinements in Inkscape (https://inkscape.org).

### ﻿Morphological study

We examined all *Alpinia* species included in this study for key morphological traits. These traits include ligule, inflorescence branching pattern, arrangement of cincinni, bracteole shape, presence of lateral staminodes, and flower sexual dimorphism. We examined traits using dried and spirit-preserved material and photographs (when available). For species with sequences sourced from GenBank, we obtained morphological data from voucher specimens linked to these accessions (when available and showing the relevant characters) or from protologues and descriptions in monographs (Schumann 1904; Ridley 1916; Koidzumi 1917; Burtt and Smith 1972b; Smith 1975, 1978, 1991a).

### ﻿Distribution mapping

We extracted occurrence data from voucher specimens represented in the phylogenetic analysis and supplemented this with occurrence data from additional voucher specimens of the same species, even when those specific specimens were not included in the phylogenetic tree (Suppl. material 1). We georeferenced locations for specimens lacking precise occurrence data based on locality information provided on voucher labels. Using the *elevatr* package in R, we retrieved elevation data to generate a base map. We mapped species distributions using the *sf* (Pebesma 2018) and *ggplot2* (Tyner et al. 2017) packages by filtering relevant specimen records, converting coordinates into spatial features, and overlaying these points onto the elevation map.

### ﻿Typification

We generally consulted Smith (1975, 1977, 1978, 1990a), Turner (2000), and Cecchi et al. (2022) for typification of the relevant taxa, following The Code (Art. 9.11–12, Shenzhen Code) and the interpretation of Turland (2019). In the references above, Smith most often listed the types without specifically designating lectotypes, but as this is before 2001, this is to be considered lectotypification. In cases where Smith did not specify the herbarium of the type or mentioned more than one, we designate the lectotype here.

## ﻿Results

### ﻿Molecular data

We successfully sequenced 70 ITS sequences of *Alpinia* and retrieved 229 additional *Alpinioideae* sequences and two *Siphonochilus* sequences from Dryad (Suppl. material 4), resulting in lengths ranging from 411 to 688 base pairs (bp) (Suppl. material 2). Notably, 13 accessions lacked the 5.8S region sequence. The alignment of the ITS sequences from the 301 accessions resulted in a 735 bp data matrix, with detailed characteristics provided in Table 1. The lengths of the ITS1, 5.8S, and ITS2 regions ranged from 82–250 bp, 3–158 bp, and 147–236 bp, respectively. Numbers at the low ends are due to missing data, mainly in sequences from GenBank (which often excluded most of 5.8S). The average GC content is 56.01%, ranging from 52%–62%. Additionally, the number of parsimony-informative sites was higher in the ITS2 region (184 sites) compared to the ITS1 and 5.8S regions (Table 1). The number of indels is higher in the ITS2 region than in the ITS1, with sizes ranging from 1–5 in ITS1 and 1–8 in ITS2 (Table 1).

**Table 1.** Statistics describing DNA sequence alignments used for phylogenetic reconstruction of *Alpinia*. PIS = parsimony informative sites; Ts/Tv = transition/transversion ratio; ingroup 1 = Alpinioideae; ingroup 2 = Carolinensis clade.

	ITS1	5.8S†	ITS2	Total
Aligned length	283	170	282	735
Length average	208	150	217	606
Length average (ingroup 1)	208	150	217	606
Length average (ingroup 2)	234	154	217	649
Length range	82–250	3–158	147–236	411–688
Length range (ingroup 1)	82–250	3–158	147–236	411–688
Length range (ingroup 2)	137–249	3–157	161–224	421–688
GC content range (%)	49–62	33–67	53–66	52–62
GC content range (ingroup 1)	49–62	33–67	53–66	52–62
GC content range (ingroup 2)	51–60	50–67	59–64	55–59
GC content average	54.37	51.10	60.15	56.01
GC content average (ingroup 1)	54.39	51.10	60.18	56.03
GC content average (ingroup 2)	53.90	51.72	61.77	56.31
Number of indels	24	2	28	55
Size of indels	1–5	1–7	1–8	1–8
Number of indels (ingroup 1)	24	2	28	55
Size of indels (ingroup 1)	1–5	1–7	1–8	1–8
Number of indels (ingroup 2)	3	2	3	8
Size of indels (ingroup 2)	1–2	1–7	1–7	1–7
Number of PIS (total)	152	31	184	367
Number of PIS (ingroup 1)	147	29	181	357
Number of PIS (ingroup 2)	26	4	32	62
Transition/transversion ratio	1.1	3	1.37	1.28
Ts/Tv ratio (ingroup 1)	1.15	3	1.32	1.26
Ts/Tv ratio (ingroup 2)	1.41	1	3.45	2.17

†13 accessions lacked the 5.8S region in their sequences.

### ﻿Phylogenetic inference

**Figure 1.**
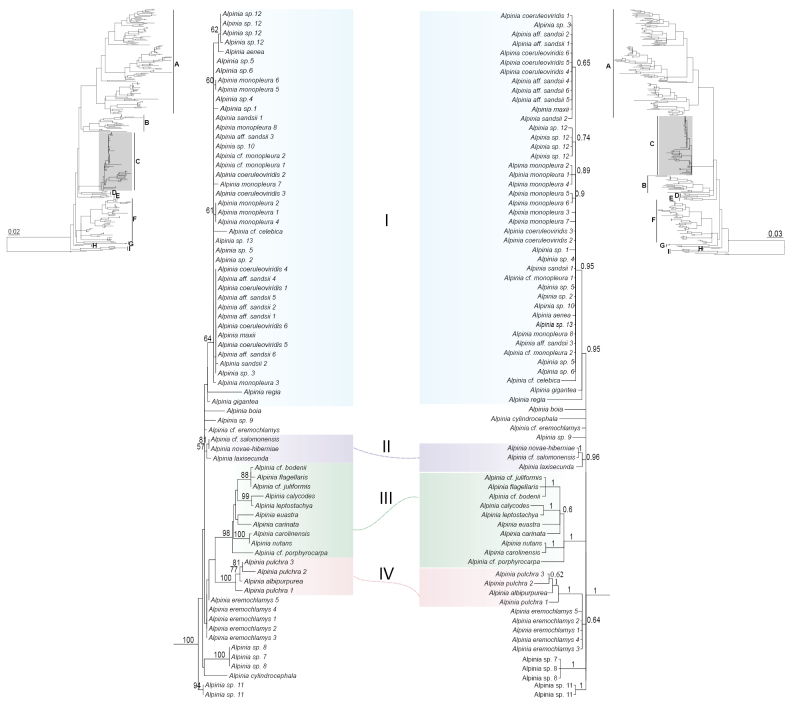
Phylogenetic relationships of the *Alpinia* “Carolinensis” clade inferred from the internal transcribed spacer (ITS) dataset. The inset presents the *Alpinioideae* phylogram with a zoomed-in view of the Carolinensis clade (for *Alpinioideae* phylograms with tip labels, see Suppl. material 3). **Left**: Maximum likelihood tree with bootstrap values (>50%) annotated on the branches. **Right**: Bayesian inference consensus tree with posterior probabilities (>0.5) annotated on the branches. A–I in the inset represent major clades, namely A: a group including *Sundamomum*, *Conamomum*, *Sulettaria*, *Geocharis*, *Wurfbainia*, *Meistera*, *Hornstedtia*, *Alpinia* “Eubractea” clade, and *Etlingera*; B: *Alpinia* “Zerumbet” clade; C: *Alpinia* “Carolinensis” clade; D: *Lanxangia* and *Geostachys*; E: *Alpinia* “Rafflesiana” clade; F: a group including *Amomum*, *Elettaria*, *Aframomum*, and *Renealmia*; G: *Alpinia* “Galanga” clade; H: *Siliquamomum*; and I: *Riedelieae*.

Phylogenetic analyses of the ITS data confirmed the presence of a well-supported Carolinensis clade (posterior probability [PP] = 1, bootstrap support [BS] = 100; see Fig. 1). The resolution within the clade was, however, insufficient to fully clarify its internal evolutionary relationships. Despite this limitation, both Bayesian and ML analyses identified several strongly supported subclades (Fig. 1), indicating significant evolutionary divergence within the Carolinensis clade. The position of the Carolinensis clade within the *Alpinioideae* lineage is consistent in both ML and Bayesian analyses, forming a polytomy with (1) clade D (*Lanxangia* and *Geostachys*) and (2) a large clade consisting of clade A (a group including *Sundamomum*, *Conamomum*, *Sulettaria*, *Geocharis*, *Wurfbainia*, *Meistera*, *Hornstedtia*, *Alpinia* “Eubractea” clade, and *Etlingera*) and clade B (*Alpinia* “Zerumbet” clade). In the Bayesian analysis, clade B forms its own distinct group within the polytomy, separating from clade A. This distinction is not seen in the ML analysis, where clade B remains part of the larger clade with clade A.

### ﻿Subclade I (PP = 0.95)

**Figure 2.**
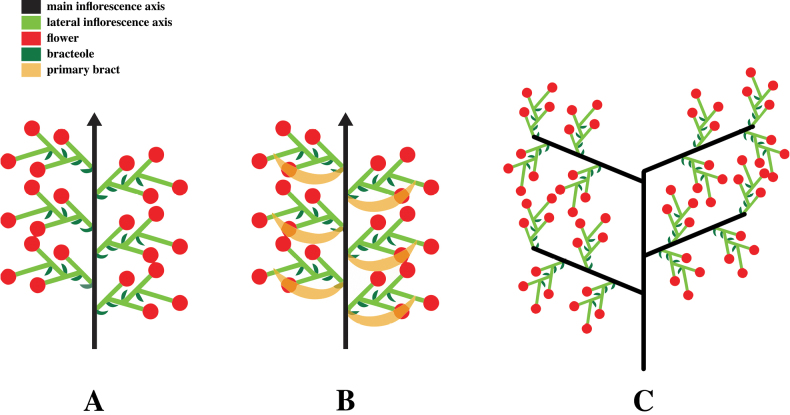
Inflorescence branching patterns observed in the Carolinensis clade. **A.** Thyrse: an inflorescence with a raceme primary axis and zigzag monochasial cyme (cincinnus) lateral branches; primary bracts are absent or minute; **B.** Thyrse inflorescence with persistent primary bracts; **C.** Secondarily branched thyrse (panicle in some terminology): a raceme primary axis with second branches and zigzag monochasial cyme lateral branches; primary bracts are absent or minute.

**Figure 3.**
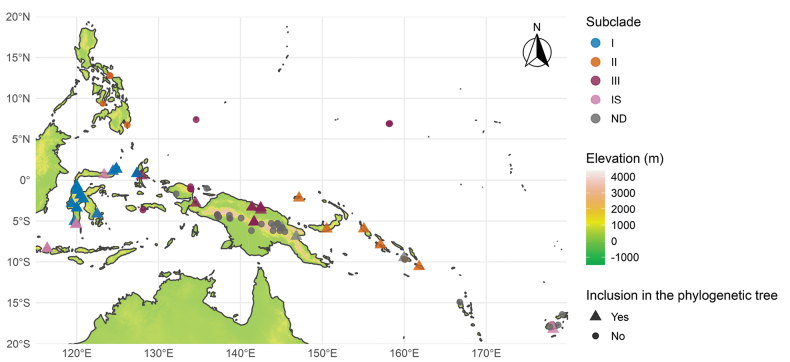
Distribution of species of the Carolinensis clade. I–IV: Subclades I–IV, IS: *Incertae Sedis*, ND: Not defined (species observed morphologically but without molecular data).

This subclade is strongly supported only in the Bayesian analysis but not in the ML analysis. Nonetheless, both analyses showed consistent topology, with *A. regia* R.M.Sm. and *A. gigantea* Blume forming a polytomy with a group comprising the remaining accessions. These accessions formed a clade strongly supported in the Bayesian analysis (PP = 0.95) but weakly supported in the ML analysis (BS < 50). This subclade, comprising 41 accessions, represents a group of *Alpinia* primarily characterized by thyrse inflorescences and reduced or absent primary bracts (Fig. 2A). The clade encompasses a range of growth forms, from large-stature species like *A. monopleura* K.Schum. to smaller species such as *A. sandsii* R.M.Sm. (Table 2). While most species display a monoecious sexual system, there are exceptions, such as *Alpinia* sp. 12. Geographically, the majority of species in this subclade are found in Sulawesi, whereas *A. gigantea* and *A. regia* are found in the Maluku Islands (Moluccas) (Fig. 3).

**Table 2.** Morphological comparison of four Carolinensis subclades.

Character	Subclade I	Subclade II	Subclade III	Subclade IV
Plant stature	large (>3 m), small (0.5–3 m)	large (>3 m)	large (>3 m), small (0.5–3 m)	large (>3 m), small (0.5–3 m)
Ligule	entire, bilobed, or emarginate	entire	entire or bilobed	bilobed
Inflorescence branching pattern	open thyrse	open thyrse	open thyrse	closed and secondarily branched thyrse
Cincinni arrangement in primary inflorescence axis	alternate and spirally arranged, alternate and distichous, or secund	alternate and spirally arranged or secund	alternate, alternate and distichous, or not secund	alternate, verticillate, or not secund
Primary bracts	absent or minute	absent or minute	large, persistent	absent or minute
Bracteole shape	tubular	tubular	tubular	tubular
Calyx	tubular with tri-lobed or truncate apex	tubular with tri-lobed or truncate apex	tubular with tri-lobed often elongated filiform apex	tubular with tri-lobed apex
Dorsal corolla lobe	cucullate	cucullate	cucullate	cucullate
Lateral staminodes	present (tooth-like) or absent	present (tooth-like)	present (tooth-like)	absent, but there are tooth-like structures at the apex of filament
Labellum	oblong	oblong	oblong	linear
Filament length	1–12 mm	1–7 mm	0–15 mm	5–15 mm
Flower sexual dimorphism	present or absent (hermaphrodite)	present	absent (hermaphrodite)	absent (hermaphrodite)
Distribution	Sulawesi, Mollucas	Bismarck Archipelago, Philippines†, Solomon Islands	New Guinea, Moluccas, Caroline Islands	New Guinea, Solomon Islands

† We did not incorporate the sequence data of the Philippine specimens in this study, but we conducted an analysis using unpublished sequence data of Philippine accessions to confirm the position of those specimens in the tree.

### ﻿Subclade II (PP = 0.96, BS = 57)

This subclade is moderately supported in the ML analysis and strongly supported in the Bayesian analysis. The species within this subclade are distributed in the Bismarck Archipelago and Solomon Islands. Morphologically, this clade shares similarities with subclade I, featuring large-statured herbs with thyrse inflorescences and reduced or minute primary bracts (Fig. 2A).

### Subclade III (PP = 1, BS = 98)

This subclade is well supported in both Bayesian and ML analyses. Bootstrap support values indicate strong to moderate confidence in the relationships within the subclade, with certain subgroups exhibiting high support, while the broader grouping shows varying levels of support (Fig. 1). Morphologically, species within this subclade are distinct from other members of the Carolinensis clade, characterized by persistent and relatively large primary bracts (Fig. 2B). The distribution of the species spans mainland New Guinea, the Moluccas, and the Caroline Islands (Fig. 3).

### ﻿Subclade IV (PP = 1, BS = 100)

This subclade is strongly supported in both Bayesian and ML trees. In the Bayesian tree, it groups with *A. eremochlamys* K.Schum. with moderate support (PP = 0.64). In the ML analysis, subclade IV is sister to subclade III, and together they form a sister group to *A. eremochlamys*, though with weak support (BS < 50). The two species within the subclade are native to New Guinea and the Solomon Islands (Fig. 3) and share distinctive morphological traits, such as the presence of tooth-like structures at the apex of the filament (Table 2).

### ﻿Incertae Sedis (no subclade)

The incertae sedis (IS) in the phylogenetic analysis include *A. cf. eremochlamys*, *A. cylindrocephala* K.Schum., *Alpinia* sp. 7, *Alpinia* sp. 8, *Alpinia* sp. 9, and *Alpinia boia* Seem. These taxa do not form part of any specific subclade within the Carolinensis lineage.

### ﻿Sister group relationships

The tree topologies inferred from ML and BI analyses differ in the suggested sister group relationships. No definitive sister group relationships were identified in the Bayesian tree, with the subclades and IS represented as polytomies. In contrast, the ML tree placed *Alpinia* sp. 11 as sister to the remaining Carolinensis lineage, albeit with weak support.

### ﻿Morphological features of the Carolinensis clade

**Figure 4.**
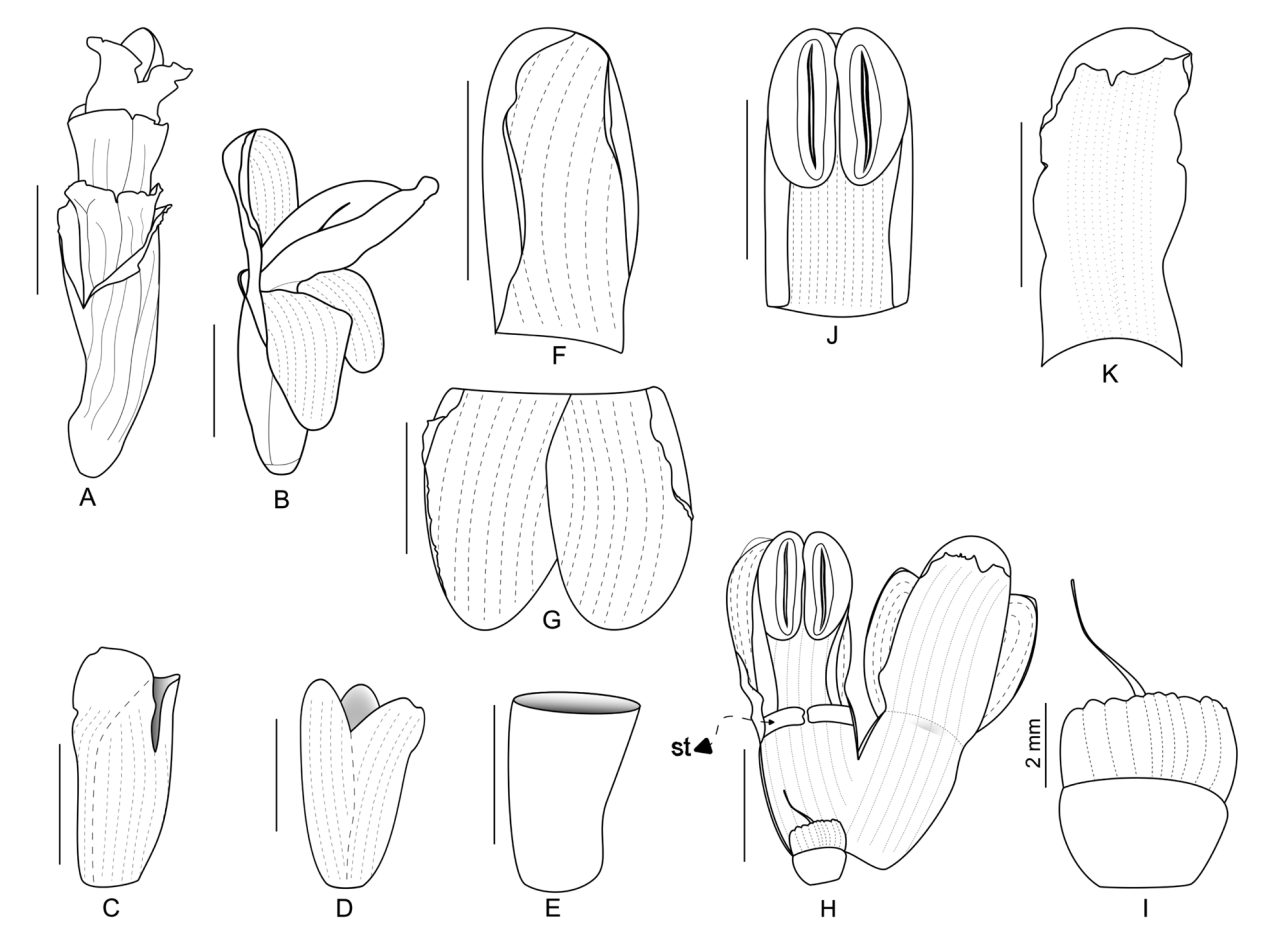
Illustration of *Alpinia* sp. **A.** Cincinnus; **B.** Male flower; **C.** Bracteole; **D.** Calyx; **E.** Floral tube; **F.** Dorsal corolla lobe; **G.** Lateral corolla lobes; **H.** Dissected male flower; **I.** Ovary, epigynous glands, and aborted style; **J.** Stamen; **K.** Labellum. St: lateral staminode. Scale bars: 1 cm unless otherwise indicated. (Drawn by S.K. Senjaya from flowers in spirit of Senjaya & al. 37 (BO)).

We examined the morphological features of the Carolinensis clade based on the new circumscription presented here, focusing on traits shared by all members and those distinctive within the group (Table 2). All members of Carolinensis display features previously considered typical of *Alpinia*, including inflorescences that are terminal to the leafy shoot, although the inflorescence in a few species is subterminal (e.g., *Alpinia aff. sandsii* voucher specimens: Poulsen & al. 2777 (BO, E); Senjaya & al. 49 (BO, CEB)). Shared traits across the clade include persistent tubular bracteoles (Fig. 4C), cucullate dorsal corolla lobes (Fig. 4F), and flowers arranged densely in a cincinnus, resulting in overlapping bracteoles that are easily recognizable (Fig. 4A). This feature was observed in all species included in this study except for *A. albipurpurea* (P.Royen) R.M.Sm., which is also unusual in having a blackish purple-tipped labellum. The inflorescence type of the group is a thyrse, as seen in many *Alpinia* in other clades (Fig. 2). However, members of subclade IV (excluding *A. albipurpurea*) exhibit a secondarily branched thyrse (sometimes called a panicle) inflorescence. Persistent and large primary bracts are observed exclusively in subclade III, and the variations in the arrangement of cincinni along the inflorescence axis are documented (Table 2).

## ﻿Discussion

### ﻿*Hellwigia* Warb.

The study of the *Alpinia* “Carolinensis” clade has hitherto been significantly constrained by limited taxon sampling, leaving the diversity of this group poorly understood, though initial studies based on a restricted number of species suggested that the Carolinensis is monophyletic (Kress et al. 2005, 2007). Species with similar geographic distributions and shared morphological traits were suspected as members of this clade. While later research supported the monophyly of the Carolinensis (de Boer et al. 2018), the issue of insufficient taxon sampling continues to hinder a comprehensive understanding of its evolutionary history.

Our study significantly expands the taxon sampling by approximately fivefold, further supporting the monophyly of the Carolinensis within the *Alpinioideae* lineage. Accordingly, we refine the taxonomic status of this clade by formally recognizing it as a distinct genus and reinstating the name *Hellwigia* Warb. for this group. Warburg (1891) originally described *Hellwigia* with a single species, *H. pulchra*. Schumann (1904) made the combination *A. pulchra* (Warb.) K.Schum., thus synonymizing the genus under *Alpinia*, and subsequent authors followed this treatment. As the present study confirms that *A. pulchra* belongs within the Carolinensis clade, we here apply the generic name *Hellwigia* for this lineage.

Based on our findings, *Hellwigia* comprises species distributed east of Huxley’s biogeographic line (Huxley 1868), ranging from Sulawesi to the West Pacific Islands (Fig. 3). Our findings align with the previously known geographical distribution of the Carolinensis clade (Kress et al. 2007), with additional species now identified in mainland New Guinea and Nusa Tenggara. Morphologically, *Hellwigia* is characterized by densely arranged flowers borne in a zigzag monochasial cyme (cincinnus), forming a thyrse or secondarily branched thyrse inflorescence. This study expands the recognized diversity of the clade by including both large-statured species from Sulawesi and the West Pacific Islands, as well as small-statured species from Sulawesi and mainland New Guinea.

### ﻿Subclades within *Hellwigia*

With the increased sampling of this study, we are now able to identify four distinct groups within *Hellwigia* (subclades I–IV) that do not fully correspond to previous classifications based on bracteole shape (Schumann 1904) or a combination of morphological traits (Smith 1990a). Although our results do not fully resolve all parts of the tree, these subclades form a basis for future study, leaving relatively few species without a subclade as *incertae sedis*.

Subclade I partially corresponds to the previously recognized section Myriocrater sensu Smith (Smith 1990a), which was characterized by a monoecious reproductive system (Burtt and Smith 1972b, Smith 1977, 1990a). However, Myriocrater does not represent a monophyletic group, as certain species, such as *A. eremochlamys* and *A. laxisecunda* B.L.Burtt & R.M.Sm., fall outside this subclade. Moreover, the monoecious trait, once considered a key character of *Myriocrater*, is inconsistent across subclade I. For instance, *Alpinia* sp. 12 displays a hermaphroditic sexual system, highlighting variability in reproductive traits within the group.

The extremely short branches observed in this subclade (I) reflect minimal character changes in the ITS dataset, potentially due to recent divergence events or the lower evolutionary rate of the ITS region in the subclade. This limitation prevents the ITS marker from resolving shallow phylogenetic relationships. Such challenges reflect the fact that ITS is unsuitable for resolving phylogenetic signal at very deep or very shallow resolution, but despite these problems (Álvarez and Wendel 2003), it has proved highly valuable at an intermediate level of resolution.

Subclade II includes species morphologically classified under section Myriocrater and distributed across the Philippines, Bismarck Archipelago, and Solomon Islands. Note that in the occurrence distribution map (Fig. 3), we added occurrence data of *A. musifolia* Ridl. and *A. vulcanica* Elmer specimens from the Philippines; we did not incorporate the sequence data of those species, but another analysis including them has confirmed their position in subclade II (Rudolph V. A. Docot, pers. comm.).

Subclade III comprises species morphologically classified within Alpinia section Dieramalpinia (Smith 1990a). This section, however, is not monophyletic, as species like *A. oceanica* Burkill and *A. vittata* W.Bull are positioned within the Eubractea clade (Kress et al. 2005, 2007). Despite its non-monophyletic nature, the defining traits of *Dieramalpinia* – unbranched (thyrse) inflorescences and large, persistent primary bracts – are reliable within the context of this clade for distinguishing subclade III from other subclades within *Hellwigia*. Bootstrap support values indicate strong to moderate confidence in the relationships within subclade III, although some internal relationships remain unresolved. This highlights the need for further investigation to clarify these relationships.

Subclade IV is a small group distinguished by the presence of *Hellwigia* species with secondarily branched thyrse inflorescences, historically referred to as paniculate in earlier taxonomic literature. These species align with Alpinia section Pycnanthus subsection Pycnanthus (Smith 1990a). The placement of *A. boia*, another species with a secondarily branched thyrse inflorescence, outside this subclade suggests that this inflorescence type does not define a distinct evolutionary lineage within the clade. This subclade also includes *A. albipurpurea*, which differs morphologically by having flowers arranged in loose cincinni or possibly as single flowers. Although only one species with this floral trait was included in the present phylogenetic study, other species such as *A. athroantha* Valeton and *A. odontonema* K.Schum. have been observed and previously reported to bear single flowers.

Several species do not fall within any identified subclade and are referred to as *incertae sedis*. Their ambiguous placement suggests that they may represent distinct lineages that diverged independently from other groups and that the current sampling and limited molecular data, including only the ITS region, are insufficient to clarify their phylogenetic relationships accurately at present.

### ﻿Distribution

*Hellwigia* is distributed east of Huxley’s Line only, a variant of Wallace’s Line proposed by Huxley (1868) separating the Philippines from Borneo. This biogeographic boundary accurately delineates the clade’s range, with no evidence of its members occurring further west. These east-west biogeographic lines in Malesia are generally supported (van Welzen et al. 2005), even though Wallacea may best be treated as a transition zone rather than a strict boundary (van Welzen et al. 2005). The absence of this clade from western Malesia may be attributed to migration barriers or benign avenues of dispersal in the past. Alternatively, as suggested by Joyce et al. (2021), environmental variables may play a stronger role in shaping the distribution patterns of this group, significantly influencing modern phytogeographic patterns.

*Hellwigia* spans four bioregions: Malesia (Philippines, Sulawesi, the Lesser Sunda Islands, the Moluccas), Papuasia (New Guinea, the Bismarck Archipelago, the Solomon Islands), Northwestern Pacific (Caroline Islands), and Southwestern Pacific (Fiji) (Fig. 3). The distribution of the identified subclades (I–IV) is quite distinct, with minimal overlap. Subclade I is found in the area called ‘reduced’ Wallacea (Darlington 1957), covering Sulawesi and Moluccas. Subclade II exhibits an intriguing distribution from the Philippines to the Bismarck Archipelago and Solomon Islands. This Pacific Rim pattern may reflect the assumed eastward dispersal of biota in Malesia, resulting in Malesia being the source of the tropical South Pacific lineages (van Balgooy 1971, Keppel et al. 2009). The same theory can also account for subclades III and IV distribution patterns. However, the apparent absence of subclade II from mainland New Guinea requires further explanation. One hypothesis for the absence in New Guinea is that extinction may have significantly shaped modern phytogeographic patterns (Keppel et al. 2009).

Members of *Hellwigia* are commonly submontane and montane in elevation. The Sulawesi species are largely montane, typically found at elevations ranging from 740 to 2,950 m. On other islands, including small ones like Ternate, species remain confined to montane habitats, e.g., *A. regia* in Mount Gamalama (Ardiyani 2010). Although some species, such as *A. porphyrocarpa* and *A. carinata*, are reported from low elevation (Smith 1975), their occurrences are restricted to valleys within the Papua Pegunungan mountain range, further emphasizing the genus’s overall association with submontane and montane environments.

### ﻿Morphological traits: inflorescence and sexual system

Inflorescence branching patterns and the presence or absence of primary bracts correlate with subclade clustering. However, the terminology for inflorescence diversity is often inconsistent, and attempts to standardize it have led to additional confusion (see Prenner et al. 2009, Endress 2010, Kirchoff and Claßen-Bockhoff 2013). To avoid ambiguity, we provide detailed descriptions of observed branching patterns rather than relying solely on terminology. We identified two branching patterns: (1) thyrse – a compound inflorescence with lateral zigzag monochasial cyme branches on a raceme primary axis, and (2) secondarily branched thyrse (sometimes called panicle in taxonomic literature) – a compound inflorescence with a raceme primary axis, secondary axes, and lateral zigzag monochasial cyme branches. The secondarily branched thyrse features a racemose branching system, where flowers are replaced by racemose partial inflorescences of the second order, resembling a double raceme (Endress 2010), but with the presence of a cyme as the third-order inflorescence in this case. Variation in primary bracts is notable, with many species having minute primary bracts, while in members of subclade III, they are large and persistent. The presence or absence of flower-subtending bracts has been identified in other monocots as a significant taxonomic marker and may contribute to the vascular organization between lateral flowers and the inflorescence axis (Remizowa et al. 2013). Despite the correlation of these traits to subclade clustering, their correlation with the evolution of *Hellwigia* remains unclear, as the functional importance of these traits is not well documented.

Floral sex dimorphism is another trait linked to subclade clustering, prominently observed in subclades I (excluding *Alpinia* sp. 12) and II. This trait was first noted by Valeton and later documented by Burtt and Smith (1972b), who used it as a defining trait for section Myriocrater (Smith 1977, 1990a). However, detailed studies on the variability and functionality of this trait within the group remain lacking. Smith (1977) noted that in *Myriocrater*, typically only the first flower – and occasionally the second flower – in a cincinnus produces fruit. Her observations of *A. regia* provided strong evidence that the development of the cincinnus in this section is delayed until fruit has been set by the first flower (Smith 1977). Our field observations corroborate this finding, and we suspect that plants within a population may function as either male or female at any given time. However, as our study did not focus on population-level dynamics, further research is required to investigate the extent and implications of this functional dimorphism in greater detail.

### ﻿Future research directions

Although the ITS region has proven useful for delineating *Hellwigia*, it has significant limitations in fully resolving its phylogenetic relationships. To overcome these challenges, future studies should adopt more robust molecular techniques, such as Hyb-Seq (Weitemier et al. 2014). Several probe sets are now available for such an approach, including the universal Angiosperm353 (Johnson et al. 2019) and the Zingiberales-specific Zingiberaceae1180 probe set (Carlsen et al. 2018). Notably, the Zingiberaceae1180 probe set has been successfully applied in other ginger lineages (Hlavatá et al. 2023; Poulsen et al. 2024) and shows great promise for elucidating evolutionary relationships within ginger lineages. By leveraging these advanced methods, future research can build on the foundational work established with ITS sequencing, offering a more comprehensive understanding of the evolutionary history of the genus.

### ﻿Species without molecular data

The phylogenetic analysis included 42 species of *Hellwigia*, of which only 31 were identified to the species level. In addition, we assign below 45 species lacking molecular data to *Hellwigia* based on their morphological characters and overlapping geographical distributions. Although these species have not been included in previous molecular phylogenetic studies, their diagnostic features closely align with those of *Hellwigia* as currently circumscribed. One notable group – species with secondarily branched thyrse inflorescences – appears closely related to *H. pulchra*. This morphological affinity has been noted before. Ridley (1916) referred to Alpinia (Hellwigia) kermesina, a synonym of *A. divaricata* Valeton. Likewise, Smith (1978) provided a detailed account of species within section Pycnanthus, which she describes as bearing paniculate inflorescences, and also placed section Monanthocrater Valeton, whose members typically bear solitary flowers. While we were initially cautious about including species with single flowers, the inclusion of *A. albipurpurea* in our phylogenetic tree supports the inclusion of morphologically similar species such as *A. athroantha*, *A. odontonema*, and *A. singuliflora* R.M.Sm. within the *Hellwigia* framework. Moreover, we confirm the placement of *A. leptostachya* Valeton within the Carolinensis clade, further justifying the inclusion of species with comparable floral morphology. Smith (1975) noted that the assignment of species such as *A. leptostachya*, *A. manostachys* Valeton, *A. gracillima* Valeton, and *A. dekockii* Valeton to subsection Kolowratia by Valeton would likely have changed if he had seen material of *A. elegans* (C.Presl) K.Schum. Smith noted that these species are closely related to, and in some cases may be identical with, species of *Eriolopha* Ridley.

In comparison to previously recognized classification (Smith 1990a), *Hellwigia* comprises species formerly assigned to Alpinia subgenus Dieramalpinia, specifically those placed in sections *Myriocrater*, *Pycnanthus*, and the majority of species in section Dieramalpinia. However, several species historically assigned to section Dieramalpinia are excluded from *Hellwigia*, as previous studies have demonstrated that they belong to other clades (Kress et al. 2007; Docot et al. 2019b). These include *A. oceanica* Burkill, *A. vittata* W.Bull, *A.* “*luteocarpa*” Elmer, *Adelmeria gigantifolia* (Elmer) Elmer, *Adelmeria oblonga* Merr., and *Adelmeria paradoxa* (Ridl.) Merr. Additionally, we refrain from proposing taxonomic treatment for two species that are morphologically ambiguous and lack molecular data, namely *A. macrocephala* K.Schum. (section Pycnanthus) and *A. condensata* Valeton (section Dieramalpinia).

## ﻿Conclusion

Molecular and morphological data support the Carolinensis clade as a distinct evolutionary lineage. Based on this evidence, we reinstate *Hellwigia* Warb. as the generic name for this clade. We recognize 76 species within *Hellwigia* and designate 26 lectotypes. The revised generic circumscription also includes 14 heterotypic synonyms; however, we acknowledge that future studies may reveal that some of these names need to be reinstated. Although several well-supported subclades are present within *Hellwigia*, the available evidence does not justify their recognition as separate genera. In particular, subclade I lacks strong phylogenetic support, and the presence of morphologically similar *incertae sedis* further complicates the subdivision of *Hellwigia*. Recognition of these subclades at the generic level would likely result in an unstable taxonomic framework. Therefore, we believe the genus-level circumscription presented here offers greater taxonomic stability. Importantly, all species within this monophyletic group share one or more consistent morphological characters that, although not exclusive to the group, are diagnostic and reinforce their inclusion in *Hellwigia*. The following is the generic circumscription of *Hellwigia*, with type specimens physically examined marked with “!” and those reviewed solely through digital scans indicated with “*.” The code inside the square brackets following the herbarium code is the barcode, unless stated otherwise.

### ﻿Taxonomy

#### ﻿ Hellwigia

Taxon classificationPlantaeZingiberalesZingiberaceae

Warb., Bot. Jahrb. Syst. 13: 279 (1891), emend. Seni Senjaya & Axel D. Poulsen

1865F06F-2143-52CE-B4C2-15BEA54FF108

Fig. 5

Eriolopha Ridl., Hook. Ic. P1. t. 3067 1916.

##### Type.

*Hellwigia pulchra* Warb., Bot. Jahrb. Syst. 13: 279 1891.

##### Description.

Small- to large-sized herb, ± clump-forming. Leaves distichously arranged, sessile to long-petiolate. Inflorescence terminal to the leafy shoot (rarely subterminal); peduncles often long, pendulous, or ascending to erect. Inflorescence architecture a thyrse, with a racemose main axis and zigzag monochasial cyme lateral branches, or a secondarily branched thyrse, with secondary branches also bearing such cymes. In some species (e.g., *A. calycodes*, *A. euastra*), cincinni congested and appearing involucre-like, though overall structure remains thyrsoid. Cincinni sessile to pedunculate arranged secund, spirally alternate, alternate and distichous, or subverticillate along the main or secondary axes. Flowers usually densely arranged within cincinni; solitary in some species (e.g., *A. albipurpurea*). Primary bracts (cincinnus bracts) either small and inconspicuous or seemingly absent, or large, brightly colored, often obscuring the main axis, and typically persistent. In species with small or absent primary bracts, inflorescence bracts usually large, papery, and caducous. Bracteoles always tubular. Flowers sessile or pedicellate; bisexual or dimorphic, with both hermaphrodite and male flowers on the same plant. Calyx narrowly or distinctly 3-lobed, when distinctly lobed, often elongated. Corolla with dorsal lobe almost always cucullate. Labellum linear or narrowly oblong, occasionally more or less triangular. Lateral staminodes present or sometimes absent. Filament usually well-developed, sometimes bearing two subapical tooth-like structures; anther may be sessile. Connective appendage sometimes crested; in dimorphic species, the crest is present on hermaphrodite flowers and absent in male flowers. Stigmas erect and more or less obconical, with a flattened ventral surface bearing a centrally positioned, narrow, horizontal elliptic orifice. Occasionally clavate, with a rounded orifice subapically or ventrally; rarely geniculate, with a small terminal orifice. Epigynous glands always large, splitting irregularly. Fruit typically globose, occasionally obovoid, and rarely pyriform.

##### Distribution.

At least 76 species (previously recognized as *Alpinia*) distributed in the Philippines, Sulawesi, the Caroline Islands, the Lesser Sunda Islands, the Moluccas, Papua, the Bismarck Archipelago, the Solomon Islands, and the Southwest Pacific Islands (Fiji, Samoa, and Vanuatu) (Fig. 3). The greatest diversity of *Hellwigia* is found in the islands of Sulawesi and Papua.

##### Etymology.

The German botanist Otto Warburg (1859–1938) named the genus after his companion on an expedition in the former Kaiser-Wilhelmsland, currently Papua New Guinea, Franz Carl Hellwig (1861–1889), who died shortly thereafter at Finschhafen. Together, they discovered *H. pulchra* in the nearby forests above Sattelberg.

**Figure 5.**
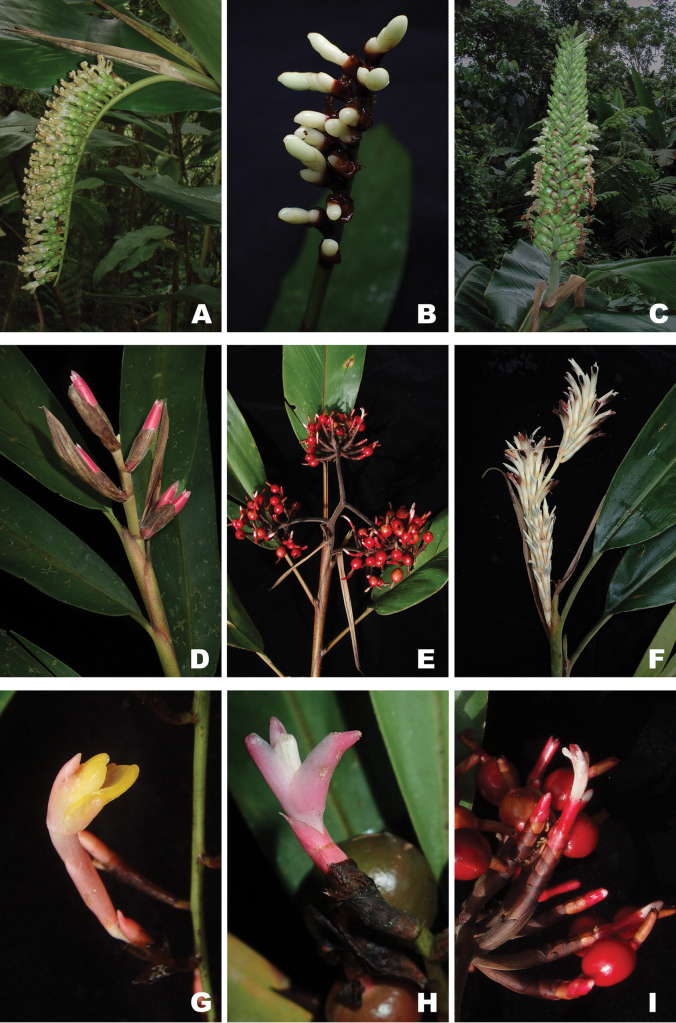
*
Hellwigia* Warb. **A.***Hellwigia laxisecunda* (inflorescence; Poulsen et al. 2482); **B.***Hellwigia coeruleoviridis* (inflorescence; Senjaya et al. 33); **C.***Hellwigia salomonensis* (inflorescence; Poulsen et al. 2581); **D.***Hellwigia leptostachya* (inflorescence; Poulsen et al. 3075); **E.***Hellwigia pulchra* (inflorescence; Poulsen 2468); **F.***Hellwigia albipurpurea* (inflorescence; Poulsen et al. 3018); **G.***Hellwigia maxii* (cincinnus; Poulsen and Firdaus 2656); **H.***Hellwigia leptostachya* (cincinnus; Poulsen et al. 3075); **I.***Hellwigia pulchra* (cincinnus; Poulsen 2468). (Photos: **A, C–I.** A.D. Poulsen; **B.** Roland P.P. Ahmad).

#### ﻿ Hellwigia acuminata

Taxon classificationPlantaeZingiberalesZingiberaceae

(RM.Sm.) Senjaya & A.D.Poulsen comb. nov.

C41A74E8-E9E4-5D22-B342-7331A06CD3F2

urn:lsid:ipni.org:names:77367765-1

Alpinia acuminata R.M.Sm., Edinburgh J. Bot. 47: 63 1990 (Basionym). Rhynchanthus papuanus Gilli, Ann. Naturhist. Mus. Wien, 84B: 46 1983. Type: Papua New Guinea. Western Highlands, A. Gilli 440 (holotype: W* [W 1979-0016650]).

#### ﻿ Hellwigia aenea

Taxon classificationPlantaeZingiberalesZingiberaceae

(B.L.Burtt & R.M.Sm.) Senjaya & A.D.Poulsen comb. nov.

35AABE69-97F6-5F45-887C-CE2D226B43ED

urn:lsid:ipni.org:names:77367766-1

Alpinia aenea B.L.Burtt & R.M.Sm. Notes Roy. Bot. Gard. Edinburgh 32: 42 1972 (Basionym). Type: Indonesia. South Sulawesi: Latimojong Mountains, M.J.S. Sands 595 (holotype: K! [K000292399–401]; isotype: E! [E00149595–596).

#### ﻿ Hellwigia albipurpurea

Taxon classificationPlantaeZingiberalesZingiberaceae

(P.Royen) Senjaya & A.D.Poulsen comb. nov.

3B3BD11A-CD47-5B11-BDA5-003A201F6117

urn:lsid:ipni.org:names:77367767-1

Kolowratia albipurpurea P.Royen, The Alpine Flora of New Guinea 2: 890 1979 (Basionym). Alpinia albipurpurea (P.Royen) R.M.Sm., Edinburgh J. Bot. 47: 64 1990. Type: Papua New Guinea. Eastern Highland: Mount Wilhelm, M. van Balgooy 583 (holotype: L! [L0041066]; isotypes: K! [K000292452], E! [E00149538]).

#### ﻿ Hellwigia arfakensis

Taxon classificationPlantaeZingiberalesZingiberaceae

(K.Schum.) Senjaya & A.D.Poulsen comb. nov.

928665B6-6C70-5E52-B85F-93E391CE7117

urn:lsid:ipni.org:names:77367768-1

Alpinia arfakensis K.Schum., Bot. Jahrb. Syst. 27: 296 1899 (Basionym). Type: Indonesia. West Papua: Arfak Mountains, O. Beccari s.n. (July 1875) (lectotype: designated by Cecchi et al., Plant Biosyst. 156(3): 781 2022. FI! [FI008297]).

#### ﻿ Hellwigia athroantha

Taxon classificationPlantaeZingiberalesZingiberaceae

(Valeton) Senjaya & A.D.Poulsen comb. nov.

FE550DCB-AE0A-55DE-88E3-40985973A798

urn:lsid:ipni.org:names:77367769-1

Alpinia athroantha Valeton, Nova Guinea 8: 952 1913 (Basionym). Type: Indonesia. Southwest New Guinea, G.M. Versteeg 1438 (lectotype: designated here, K! [K000292450-451]; isolectotype: BO! [Sheet No. BO-0080938], E! [E00504284-285]).

##### Note.

The superior condition of the duplicate at K is the reason for our choice of lectotype.

#### ﻿ Hellwigia biakensis

Taxon classificationPlantaeZingiberalesZingiberaceae

(R.M.Sm.) Senjaya & A.D.Poulsen comb. nov.

B7CD4645-2EF1-5B1B-8AC0-437851286265

urn:lsid:ipni.org:names:77367770-1

Alpinia biakensis R.M. Sm., Notes Roy. Bot. Gard. Edinburgh 35: 2015 1977 (Basionym). Type: Indonesia. Papua: Biak Islands, A.J.G.H. Kostermans & Soegang 882 (holotype: L! [L 0041067-068]).

#### ﻿ Hellwigia boia

Taxon classificationPlantaeZingiberalesZingiberaceae

(Seem.) Senjaya & A.D.Poulsen comb. nov.

9430E74E-5D2A-5DD9-8FE8-BF30A3792A1A

urn:lsid:ipni.org:names:77367771-1

Alpinia boia Seem., Fl. Vit. 290, t.88 1868 (Basionym). Type: Fiji. Viti Levu, Seeman 620 (lectotype: designated by Smith, Notes Roy. Bot. Gard. Edinburgh 36(2): 275 1978, K! [K000928019-020]).

#### ﻿ Hellwigia calycodes

Taxon classificationPlantaeZingiberalesZingiberaceae

(K.Schum.) Senjaya & A.D.Poulsen comb. nov.

C6EC845C-4A62-5A94-ABEC-C5E91F7B8678

urn:lsid:ipni.org:names:77367772-1

Alpinia calycodes K.Schum., Bot. Jahrb. Syst. 27: 295 1899 (Basionym). Type: Indonesia. West Papua, Andai, O. Beccari 589 (lectotype: designated by Cecchi et al. Plant Biosyst. 156(3): 781 2022. FI! [FI008299]).

#### ﻿ Hellwigia carinata

Taxon classificationPlantaeZingiberalesZingiberaceae

(Valeton) Senjaya & A.D.Poulsen comb. nov.

6DE7C60F-D224-59DA-91EB-1DC6983C8912

urn:lsid:ipni.org:names:77367773-1

Alpinia carinata Valeton, Nova Guinea 8: 945 1913 (Basionym). Types: Indonesia. Papua: near Sabang and Alkmaar, Mundung River, G.M. Versteeg 1283 (lectotype: designated here, L! [L 0427768]), 1271 (syntype: BO! [Sheet No. BO-0080214–216]), n. 1285 (syntype: BO! [Sheet No. BO-0080217]), Branderhorst 368 (syntype: n.v.).

##### Note.

Valeton (1913) mentioned four syntypes, of which we consider Versteeg 1283 to be the best.

#### ﻿ Hellwigia carolinensis

Taxon classificationPlantaeZingiberalesZingiberaceae

(Koidz.) Senjaya & A.D.Poulsen comb. nov.

27AD46FB-D3D7-5A88-955C-22E7D81D467A

urn:lsid:ipni.org:names:77367774-1

Alpinia carolinensis Koidz., Bot. Mag. Tokyo 31: 233 1917 (Basionym). Languas carolinensis (Koidz.) Kaneh., Fl. Micron. 412 1933. Type: Micronesia. Ponape Island, Koidzumi s.n. (holotype: TI* [TI 00367997–999).
Languas babeldaobensis Kaneh., Trans. Nat. Hist. Soc. Formosa 25: 7 1935. Type: Palau Aimiriik, Kanehira 321 (syntype: n.v.), Kanehira 2295 (syntype: n.v.), Marikyoku 430 (syntype: n.v.).

#### ﻿ Hellwigia celebica

Taxon classificationPlantaeZingiberalesZingiberaceae

(Ridl.) Senjaya & A.D.Poulsen comb. nov.

8E3165D3-7C06-5663-9216-8E151E42EEB8

urn:lsid:ipni.org:names:77367775-1

Alpinia celebica Ridl., J. Straits Branch Roy. Asiat. Soc. 34: 98 1900 (Basionym). Type: Indonesia. Gorontalo, J.G.F. Riedel s.n. (lectotype, designated by Smith, Edin. J. Bot. 47(1): 40 1990, K! [K000292402]; isolectotype: BO! [Sheet No. BO-0080211]).

##### Notes.

Schumann (1904) also published *Alpinia celebica*, based on the same type. The duplicate at BO is recorded there as the holotype. Smith (1990a) lectotypified Riedel’s specimen at K, and here we add the isolectotype that we found at BO.

#### ﻿ Hellwigia chaunocolea

Taxon classificationPlantaeZingiberalesZingiberaceae

(K.Schum.) Senjaya & A.D.Poulsen comb. nov.

015C4449-3C6E-55CE-91F0-2ABEE20C0480

urn:lsid:ipni.org:names:77367776-1

Alpinia chaunocolea K.Schum., Bot. Jahrb. Syst. 27: 292 1899 (Basionym). Type: Indonesia. West Papua, O. Beccari 916 (lectotype: designated by Cecchi et al. Plant Biosyst. 156(3): 781 2022. FI! [FI008300]).

#### ﻿ Hellwigia coeruleoviridis

Taxon classificationPlantaeZingiberalesZingiberaceae

(K.Schum.) Senjaya & A.D.Poulsen comb. nov.

DFBA896D-1474-5B43-8069-9450915AFBA1

urn:lsid:ipni.org:names:77367841-1

Alpinia coeruleoviridis K.Schum., Bot. Jahrb. Syst., 27(3): 293 1899 (Basionym). Type: Indonesia. Sulawesi, K.F. & P.B.Sarasin s.n. (type: B n.v., assumed lost in the Second World War).

#### ﻿ Hellwigia conferta

Taxon classificationPlantaeZingiberalesZingiberaceae

(B.L.Burtt & R.M.Sm.) Senjaya & A.D.Poulsen comb. nov.

49F56332-16B6-5E10-B080-12654A6EBD0F

urn:lsid:ipni.org:names:77367777-1

Alpinia conferta B.L.Burtt & R.M.Sm., Notes Roy. Bot. Gard. Edinburgh 32: 40 1972 (Basionym). Type: South Solomons, Guadalcanal, Mt. Popomanaseu, E.J.H. Corner 107 (holotype: K! [K000928009–010], isotype: E! [E00149643], NY* [00320187]).

#### ﻿ Hellwigia conglomerata

Taxon classificationPlantaeZingiberalesZingiberaceae

(R.M.Sm.) Senjaya & A.D.Poulsen comb. nov.

42898EC8-C8E2-5784-BBB1-431FC57D87B2

urn:lsid:ipni.org:names:77367778-1

Alpinia conglomerata R.M. Sm., Notes Roy. Bot. Gard. Edinburgh 36: 277 1978 (Basionym). Type: Papua New Guinea. Eastern Highlands, P. van Royen NGF 18154 (holotype: LAE! [sheet no. 58297], isotype: E! [E00275015, E00149542]).

#### ﻿ Hellwigia cylindrocephala

Taxon classificationPlantaeZingiberalesZingiberaceae

(K.Schum.) Senjaya & A.D.Poulsen comb. nov.

C7363310-F7DF-530B-B907-6EF9AB4953A8

urn:lsid:ipni.org:names:77367779-1

Alpinia cylindrocephala K. Schum., Bot. Jahrb. Syst. 27(3): 297 1899 (Basionym). Type: Indonesia. Sulawesi, Lampobatang, K.F. & P.B. Sarasin 1243 (syntype: B n.v., assumed lost in the Second World War), O. Warburg 16888 (syntype: B n.v., assumed lost in the Second World War).

#### ﻿ Hellwigia dasystachys

Taxon classificationPlantaeZingiberalesZingiberaceae

(Valeton) Senjaya & A.D.Poulsen comb. nov.

C3F70D7F-9FC5-5826-B643-41ED18967336

urn:lsid:ipni.org:names:77367780-1

Alpinia dasystachys Valeton, Nova Guinea 8: 946 1913 (Basionym). Types: Indonesia. West Papua, L.S.A.M. von Römer 1114 (lectotype: designated here, BO! [Sheet No. BO-0080569]), 1016 (syntype: BO! [BO-0080570]), 1157 (syntype: BO! [BO-0080571]).

##### Notes.

Valeton (1913) listed three collections of von Römer (syntypes), and we choose von Römer 1114 because of its superior condition.

#### ﻿ Hellwigia dekockii

Taxon classificationPlantaeZingiberalesZingiberaceae

(Valeton) Senjaya & A.D.Poulsen comb. nov.

9875BDF5-D4BE-56A8-A4EA-576B03F5B758

urn:lsid:ipni.org:names:77367781-1

Alpinia dekockii Valeton, Nova Guinea 8: 949 1913 (Basionym). Type: Papua, Mount Goliath [Peak Yamin, Jayawijaya Mts.], A.C. de Kock 17 (lectotype: designated here, BO! [Sheet No. BO-0080572])

##### Notes.

In the protologue, Valeton (1913) cited “de Kock n. 1911,” which was previously interpreted as a collection number. The specimen housed at BO is labelled as de Kock No. 17 and also bears an annotation reading “AC. de Kock 22 M. 1911,” which appears to be the source of confusion. The de Kock No. 17 specimen, as cited in the protologue, was collected from the Goliath Mountain.

#### ﻿ Hellwigia densiflora

Taxon classificationPlantaeZingiberalesZingiberaceae

(K.Schum.) Senjaya & A.D.Poulsen comb. nov.

B2FBCA52-6CA1-577B-B483-07392FA280EB

urn:lsid:ipni.org:names:77367782-1

Alpinia densiflora K.Schum., Bot. Jahrb. Syst. 27: 292 1899 (Basionym). Type: Indonesia. West Papua, O. Beccari 916 (designated by Cecchi et al. Plant Biosyst. 156(3): 782 2022. FI! [FI008305]).

#### ﻿ Hellwigia divaricata

Taxon classificationPlantaeZingiberalesZingiberaceae

(Valeton) Senjaya & A.D.Poulsen comb. nov.

748742CF-683D-5BA8-8B24-40115E91578F

urn:lsid:ipni.org:names:77367783-1

Alpinia divaricata Valeton, Nova Guinea 8: 950 1913 (Basionym). Type: Indonesia. Papua, G.M. Versteeg 1657 (lectotype: designated here, L! [L 0360111–114]; isolectotypes: BO! [Sheet No. BO-0080573–575], K! [K000292447–448]).
Alpinia kermesina Ridl., Trans. Linn. Soc. London, Bot. 9: 214 1916. Type: Indonesia. Papua: Mount Carstensz, Camp III, C.B. Kloss s.n. Dec-Jan [1912–1913] (lectotype: designated by Smith, Edin. J. Bot. 47(1): 48 1990, K! [K000292445]; isolectotype: BM! [BM014605305]).

##### Notes.

We designated the lectotype of *H. divaricata* due to the superior quality of the L duplicate. Regarding *H. kermesina*, Smith (1990a) lectotypified C.B. Kloss’s collection from Camp III at K, and here we add the isolectotype that we found at BM.

#### ﻿ Hellwigia domatifera

Taxon classificationPlantaeZingiberalesZingiberaceae

(Valeton) Senjaya & A.D.Poulsen comb. nov.

E4CEF83D-FAA4-5D1C-B2B2-DFAED17B1FB5

urn:lsid:ipni.org:names:77367784-1

Alpinia domatifera Valeton, Nova Guinea 8: 955 1913 (Basionym). Type: Indonesia. West Papua: Arfak Mountains, K. Gjellerup 1022 (lectotype: designated by Smith, Notes Roy. Bot. Gard. Edinburgh 34(2): 175 1975, L! [L 0427797]; isolectotype: BO! [Sheet No. BO-0080585]).
Eriolopha meyeri Ridl., Trans. Linn. Soc. London, Bot. 9:218 1916. Type: Indonesia. Papua: Geelvink Bay, A.B. Meyer 10 (lectotype: designated by Smith, Notes Roy. Bot. Gard. Edinburgh 34(2): 175 1975, K! [K000292444]).

##### Note.

Smith (1990a) lectotypified K. Gjellerup’s collection at L, and here we add the isolectotype that we found at BO.

#### ﻿ Hellwigia eremochlamys

Taxon classificationPlantaeZingiberalesZingiberaceae

(K.Schum.) Senjaya & A.D.Poulsen comb. nov.

3D25122C-B012-5BA2-9D93-72A693667325

urn:lsid:ipni.org:names:77367785-1

Alpinia eremochlamys K.Schum., Bot. Jahrb. Syst. 27(3): 288 1899 (Basionym). Types: Indonesia. Southeast Sulawesi: Kendari, Beccari s.n. (lectotype: designated by Cecchi et al., Plant Biosyst. 156(3): 782 2022, FI* [FI013495]), Meyer s.n. (syntype: B n.v., assumed lost during the Second World War), K.F. & P.B. Sarasin 412 (syntypes: B* [B 810000383, flowers in the spirit]; BAS* [BAS-00000257, not examined]).
Alpinia pectinata Ridl., J. Straits Branch Roy. Asiat. Soc. 34: 97 1900. Type: Indonesia. North Sulawesi: Gunung Klabat, S.H. Koorders 19650ß (lectotype: designated by Turner and Cheek, Gard. Bull. Sing. 50: 116 1998. BO! [Sheet No. BO-0035005]).

#### ﻿ Hellwigia euastra

Taxon classificationPlantaeZingiberalesZingiberaceae

(K.Schum.) Senjaya & A.D.Poulsen comb. nov.

03422997-3B23-590C-9266-2463A4FFE050

urn:lsid:ipni.org:names:77367786-1

Alpinia euastra K.Schum., Bot. Jahrb. Syst. 27: 296 1899 (Basionym). Type: Indonesia. West Papua, O. Beccari 348 (lectotype: designated by Cecchi et al. Plant Biosyst. 156(3): 783 2022. FI! [FI008306]).

#### ﻿ Hellwigia flagellaris

Taxon classificationPlantaeZingiberalesZingiberaceae

(Ridl.) Senjaya & A.D.Poulsen comb. nov.

363981F3-B6AC-56D1-AC89-8949DF60FB5C

urn:lsid:ipni.org:names:77367787-1

Eriolopha flagellaris Ridl. Hooker’s Icon. Pl. 31: t. 3067 1916 (Basionym). Alpinia flagellaris (Ridl.) Loes. Nat. Pflanzenfam. ed. 2, 15a: 622 1930. Types: Indonesia. Papua: Mt. Carstensz, Camp VIII–IX, C.B. Kloss s.n. (28 Jan 1913) (lectotype: designated here, K! [K000292443]), C.B. Kloss s.n. (Dec 1912) (syntype: BM! [BM000617287]), Camp VIc, C.B. Kloss s.n. (BM*, specimen without a barcode).

##### Notes.

Smith (1990a) mentioned that types were deposited at BM and K, but the C.B. Kloss s.n. collection at BM has a different date from that informed by Ridley in the protologue.

#### ﻿ Hellwigia gigantea

Taxon classificationPlantaeZingiberalesZingiberaceae

(Blume) Senjaya & A.D.Poulsen comb. nov.

C10962CA-40C3-505C-A561-AF35710F0F7A

urn:lsid:ipni.org:names:77367788-1

Alpinia gigantea Blume, Enum. pl. Javae 59 1827 (Basionym). Type: Indonesia. Moluccas: Ternate, C.G.C. Reinwardt s.n. (lectotype: designated by Smith, Notes Roy. Bot. Gard. Edinburgh 35: 206 1977, L! [L 0360113–114]; isolectotype: E! [E00149605]).
Alpinia myriocratera K.Schum., Bot. Jahrb. Syst. 27: 290 1899. Type: Indonesia. Moluccas: Ternate, O. Beccari s.n. (November 1874) (lectotype: designated by Cecchi et al. Plant Biosyst. 156(3): 783 2022. FI! [FI008314]).

##### Notes.

Smith (1990a) listed *A. myriocratera* as an accepted species, whereas here we consider it a synonym. In an unpublished manuscript, however, Smith reached the same conclusion.

#### ﻿ Hellwigia glacicaerulea

Taxon classificationPlantaeZingiberalesZingiberaceae

(R.M.Sm.) Senjaya & A.D.Poulsen comb. nov.

C9C6FCDA-E0B1-58C2-BFD0-15F1816EFEFA

urn:lsid:ipni.org:names:77367789-1

Alpinia glacicaerulea R.M.Sm., Edinburgh J. Bot. 48: 349 1991 (Basionym). Type: Indonesia. Sulawesi: Roroka Timbu, M. van Balgooy 3194 (holotype: L! [L 0041069], isotypes: A* [02532347], BO! [Sheet No. BO-0087181], E! [E00149606]).

#### ﻿ Hellwigia gracillima

Taxon classificationPlantaeZingiberalesZingiberaceae

(Valeton) Senjaya & A.D.Poulsen comb. nov.

D6629A73-637F-54BE-8463-F2624AB0EB6D

urn:lsid:ipni.org:names:77367790-1

Alpinia gracillima Valeton, Nova Guinea 8: 948 1913 (Basionym). Type: Indonesia. West Papua, L.S.A.M. von Römer 763 (lectotype: designated here, BO! [Sheet No. BO-8034792]), 1108 (syntype: BO!), Exploratie-Detachement 1912 (n.v.).

##### Notes.

Valeton (1913) cited three specimens as syntypes, one of which could not be located. Among the two remaining specimens, von Römer 763 is in better condition.

#### ﻿ Hellwigia hagena

Taxon classificationPlantaeZingiberalesZingiberaceae

(R.M.Sm.) Senjaya & A.D.Poulsen comb. nov.

7320D62A-353B-5D90-9385-B1A38B3A21C3

urn:lsid:ipni.org:names:77367791-1

Alpinia hagena R.M.Sm., Notes Roy. Bot. Gard. Edinburgh 36: 284 1978 (Basionym). Type: Papua New Guinea. Western Highlands, P.F. Stevens LAE 50267 (holotype: E! [E00149545]; isotype: LAE n.v.).

##### Note.

Smith (1978) mentioned the isotype at LAE, but we were unable to locate it.

#### ﻿ Hellwigia himantoglossa

Taxon classificationPlantaeZingiberalesZingiberaceae

(Ridl.) Senjaya & A.D.Poulsen comb. nov.

F39AF632-1940-512A-8B0B-0F863D5FA66B

urn:lsid:ipni.org:names:77367792-1

Alpinia himantoglossa Ridl., Trans. Linn. Soc. London, Bot. 9: 212 1916 (Basionym). Types: Indonesia. Papua, Camp VIb, C.B. Kloss s.n. (Feb 1913) (lectotype: designated here, BM* [BM000617282]), C.B. Kloss s.n. (27 Jan 1913) (syntype: K* [K000292442]).

##### Notes.

Turner (2000) mentioned both types and we select here the BM collection because of the superior condition.

#### ﻿ Hellwigia horneana

Taxon classificationPlantaeZingiberalesZingiberaceae

(K.Schum.) Senjaya & A.D.Poulsen comb. nov.

4C5A3EB1-2D10-5B47-B9F8-53791F65BD38

urn:lsid:ipni.org:names:77367793-1

Alpinia horneana (K.Schum.), Pflanzenr. IV, 46: 349 1904 (Basionym). Type: Fiji. Horne s.n. (lectotype: designated here, K! [K00928023]).

##### Notes.

Schumann (1904) cited Horne’s s.n. specimen but did not explicitly designate the type. Later, Smith (1990a) referred to this specimen as the type but did not specify where it is deposited.

#### ﻿ Hellwigia inaequalis

Taxon classificationPlantaeZingiberalesZingiberaceae

(Ridl.) Senjaya & A.D.Poulsen comb. nov.

4A2D3D99-A454-55B4-A221-1A16064EB6F3

urn:lsid:ipni.org:names:77367794-1

Psychanthus inaequalis Ridl. Trans. Linn. Soc. London, Bot. 9: 215 1916 (Basionym). Alpinia inaequalis (Ridl.) Loes. Nat. Pflanzenfam., ed. 2. 15a: 614 1930. Type: Indonesia. Papua: Utakwa River to Mt. Carstensz, Camp VIb, C.B. Kloss s.n. (Jan 1913) (lectotype: designated here, BM! [BM000617605]; isolectotypes: K! [K000292441], E! [E00149546]).

##### Notes.

Ridley (1916) cited C.B. Kloss’s specimen from Camp VIb, and Smith (1978) later identified the specimens in K and BM as the types. Here, we designate the BM specimen as the lectotype.

#### ﻿ Hellwigia janowskii

Taxon classificationPlantaeZingiberalesZingiberaceae

(Valeton) Senjaya & A.D.Poulsen comb. nov.

DE0B28EF-CD7C-54E1-8B01-2E330A7FDCE5

urn:lsid:ipni.org:names:77367795-1

Alpinia janowskii Valeton, Nova Guinea 8: 956 1913 (Basionym). Type: Indonesia: West Papua, R.Fr. Janowski 176 (lectotype: designated here, BO! [Sheet No. BO-0081566]).

##### Note.

Valeton (1913) cited Janowski’s 176 specimens, and Smith (1990a) referred to it as the type but did not specify the herbarium where it is housed.

#### ﻿ Hellwigia juliformis

Taxon classificationPlantaeZingiberalesZingiberaceae

(Ridl.) Senjaya & A.D.Poulsen comb. nov.

4092ED5F-91B2-5B7B-9210-0604C036F1AC

urn:lsid:ipni.org:names:77367796-1

Eriolopha juliformis Ridl., Trans. Linn. Soc. London, Bot. 9: 219 1916 (Basionym). Alpinia juliformis (Ridl.) R.M.Sm., Edinburgh J. Bot. 47: 65 1990. Type: Indonesia. Papua, Mt. Carstensz, Camp VIa, C.B. Kloss s.n. (09 Jan 1913) (lectotype: designated here, BM! [BM000617289]; isolectotypes: E! [E00149666], K! [K000292440]).

#### ﻿ Hellwigia kiungensis

Taxon classificationPlantaeZingiberalesZingiberaceae

(R.M.Sm.) Senjaya & A.D.Poulsen comb. nov.

C3B2755E-3E4F-5BE4-8848-6F92FB9E10D1

urn:lsid:ipni.org:names:77367797-1

Alpinia kiungensis R.M.Sm., Notes Roy. Bot. Gard. Edinburgh 34(2): 163 1975 (Basionym). Type: Papua New Guinea. Western Province: Kiunga, H. Streimann & Y. Lelean NGF 34126 (holotype: E! [E00149671]; isotype: LAE! [Sheet No. 202360]).

#### ﻿ Hellwigia klossii

Taxon classificationPlantaeZingiberalesZingiberaceae

(Ridl.) Senjaya & A.D.Poulsen comb. nov.

5ACA1F90-2834-5DD0-A93F-D279B8B478CE

urn:lsid:ipni.org:names:77367798-1

Eriolopha klossii Ridl., Trans. Linn. Soc. London, Bot. 9: 220 1916 (Basionym). Alpinia klossii (Ridl.) R.M.Sm., Edinburgh J. Bot. 47: 65 1990. Types: Indonesia. Papua, Mt. Carstensz, Camp I-III, C.B. Kloss s.n. (22 Nov 1912) (lectotype: second step, designated here, BM* [BM000617298]), Camp III, C.B. Kloss s.n. (Jan 1913) (syntype: BM* [BM000617298].

##### Note.

Smith (1990a) mentioned the type at BM; since there are two collections by C.B. Kloss, we select the best here.

#### ﻿ Hellwigia laxisecunda

Taxon classificationPlantaeZingiberalesZingiberaceae

(B.L.Burtt & R.M.Sm.) Senjaya & A.D.Poulsen comb. nov.

95231976-0593-572F-A5DD-1B1A4DC224C5

urn:lsid:ipni.org:names:77367799-1

Alpinia laxisecunda B.L.Burtt & R.M.Sm., Notes Roy. Bot. Gard. Edinburgh 32(1): 39 1972 (Basionym). Type: Solomon Islands. San Cristobal, Sore 2316 (holotype: K! [K000928007–008]; isotypes: A* [00057373], E! [E00149645–646], L! [L 0041072–073], LAE! [Sheet No. 209982], U! [U 0007204]).

#### ﻿ Hellwigia leptostachya

Taxon classificationPlantaeZingiberalesZingiberaceae

(Valeton) Senjaya & A.D.Poulsen comb. nov.

00EADC20-6C51-574F-B969-FC2FF2E6304A

urn:lsid:ipni.org:names:77367800-1

Alpinia leptostachya Valeton, Nova Guinea 8: 947 1913 (Basionym). Type: Indonesia. Papua: Mount Resi, G.M. Versteeg 1642 (lectotype: designated by Smith, Notes Roy. Bot. Gard. Edinburgh 34(2): 175 1975, L! [L 0041074–075]; isolectotype: BO! [BO-0081755–756], E! [E00504287–288]).

#### ﻿ Hellwigia macrocarpa

Taxon classificationPlantaeZingiberalesZingiberaceae

(Valeton) Senjaya & A.D.Poulsen comb. nov.

9963816E-10EB-5641-8613-51044E6512C2

urn:lsid:ipni.org:names:77367801-1

Alpinia macrocarpa Valeton, Nova Guinea 8: 944 1913, non Gagnep. 1906 (Basionym). Alpinia valetoniana Loes., Nat. Pflanzenfam. ed. 2, 15a: 622 1930. Types: Papua, G.M. Versteeg 1270 (lectotype: designated here, L! [L0041083], U! [U0007208], K! [K000292425]), 1284 (syntype: n.v.), B. Branderhorst 365 (syntype: L! [L0041084]; isolectotype: BO! [BO-0079759–760]), von Rômer 538 (syntype: n.v.).

##### Notes.

Of the four syntypes listed by Smith (1990a), we designate G.M. Versteeg 1270 as the lectotype due to its better condition and the clear representation of the inflorescence and cincinni characters.

#### ﻿ Hellwigia manostachys

Taxon classificationPlantaeZingiberalesZingiberaceae

(Valeton) Senjaya & A.D.Poulsen comb. nov.

9C8C4E31-DC04-5E1F-846A-BFE6C9144479

urn:lsid:ipni.org:names:77367802-1

Alpinia manostachys Valeton, Nova Guinea 8: 949 1913 (Basionym). Type: Papua: Hellwig Mountain, L.S.A.M. von Römer 1152 (lectotype: designated by Smith, Notes Roy. Bot. Gard. Edinburgh 34(2): 175 1975, n.v.).

#### ﻿ Hellwigia maxii

Taxon classificationPlantaeZingiberalesZingiberaceae

(R.M.Sm.) Senjaya & A.D. Poulsen comb. nov.

67162C45-7C54-58D5-A1B3-D0479388ECCD

urn:lsid:ipni.org:names:77367803-1

Alpinia maxii R.M. Sm., Edinburgh J. Bot. 48: 350 1991 (Basionym). Type: Indonesia. Central Sulawesi: Roroka Timbu, M. van Balgooy 3271 (holotype: L! [L 0041076]; isotypes: A* [02532320], BO! [BO-0081757], E! [E00149607–608], K! [K000292395]).

#### ﻿ Hellwigia monopleura

Taxon classificationPlantaeZingiberalesZingiberaceae

(K.Schum.) Senjaya & A.D.Poulsen comb. nov.

98F5524B-F4F0-585A-88E3-7E5D009303AA

urn:lsid:ipni.org:names:77367804-1

Alpinia monopleura K.Schum., Bot. Jahrb. Syst. 27(3): 287 1899 (Basionym). Type: Indonesia. North Sulawesi: Tomohon, K.F. & P.B. Sarasin 219 (lectotype: designated here, BAS [BAS-00000063]; isolectotype: B n.v., assumed lost in the Second World War).

##### Note.

A single flower was found in the pocket herbarium of the Sarasin cousins in the BAS herbarium.

#### ﻿ Hellwigia multispica

Taxon classificationPlantaeZingiberalesZingiberaceae

(Ridl.) Senjaya & A.D.Poulsen comb. nov.

36951E73-E8C8-549C-8C8B-865E123F6511

urn:lsid:ipni.org:names:77367805-1

Eriolopha multispica Ridl., Trans. Linn. Soc. London, Bot. 9: 220 1916 (Basionym). Alpinia multispica (Ridl.) Loes., Nat. Pflanzenfam. ed. 2, 15a: 622 1930. Type: Indonesia. Papua: Mt. Carstensz, Camp VIb, C.B. Kloss s.n. (29 Jan 1913) (lectotype: second step, designated here, BM! [BM000617290]).

##### Notes.

In the protologue, Ridley (1916) mentioned another collection of C.B. Kloss from Camp VIc, which we were unable to locate. We therefore designate the collection from Camp VIb as the lectotype.

#### ﻿ Hellwigia musifolia

Taxon classificationPlantaeZingiberalesZingiberaceae

(Ridl.) Senjaya & A.D.Poulsen comb. nov.

1B8F7A9D-8AD8-54E8-AA33-502333B11CC1

urn:lsid:ipni.org:names:77367806-1

Alpinia musifolia Ridl., Leafl. Philipp. Bot. 2: 604 1909 (Basionym). Languas musifolia (Ridl.) Merr., Enum. Philipp. fl. pl. 1: 232 1923. Type: Philippines. Negros Oriental: Dumaguete, A.D.E. Elmer 9539 (lectotype: designated by Docot et al. Kew Bull. 77: 543 2022. K! [K000292468]; isolectotypes: A* [00030634], BO! [BO-0081298], BM* [BM000617269], E! [E00149622], F, FI, L! [L 0360115], G, GH, K! [K000292468], MO, NY, SING, US, Z).
Alpinia edanoi R.M.Sm., Notes Roy. Bot. Gard. Edinburgh 35: 200 1977. Type: Philippines. Negros: Mount Malbug, G. Edaño PNH 7132 (holotype: L! [L 0041077–078]; isotype: A* [0057374]).

#### ﻿ Hellwigia nidus-vespae

Taxon classificationPlantaeZingiberalesZingiberaceae

(A.Raynal & J.Raynal) Senjaya & A.D.Poulsen comb. nov.

54F8340D-2C96-55C9-A05F-60564BFAC595

urn:lsid:ipni.org:names:77367807-1

Alpinia nidus-vespae A. Raynal & J.Raynal, Adansonia 13: 63, f.2 1977 (Basionym). Type: New Hebrides [Vanuatu]. J. Raynal RNSH 16379 (holotype: P* [P00641664–665]; isotypes: A* [00030643], K! [K000928006], L! [L 0041079]).

#### ﻿ Hellwigia novae-hiberniae

Taxon classificationPlantaeZingiberalesZingiberaceae

(B.L.Burtt & R.M.Sm.) Senjaya & A.D.Poulsen comb. nov.

FC6F6D20-7C1E-501C-84E3-FCA66E0624CB

urn:lsid:ipni.org:names:77367808-1

Alpinia novae-hiberniae B.L. Burtt & R.M. Sm., Notes Roy. Bot. Gard. Edinburgh 32(1): 38 1972 (Basionym). Type: Papua New Guinea. New Ireland: Namatanai, M.J.S. Sands 857 (holotype: K! [K000292436–438]; isotypes: E! [E00149627–628], L! [L 0484212], LAE! [Sheet No. 146832, 281279]).

#### ﻿ Hellwigia nutans

Taxon classificationPlantaeZingiberalesZingiberaceae

(L.) Senjaya & A.D.Poulsen comb. nov.

2A2A7346-00F8-5E20-B6C5-CFE4B42B8B9E

urn:lsid:ipni.org:names:77367809-1

Globba nutans L., Mant. pl. 170 1771 (Basionym). Alpinia nutans (L.) Roscoe, Exot. Bot. 2: 93 1805. Tonemone malukuana C.K.Lim, Folia Malaysiana 17: 8 2016, nom. illeg. Type: Herb. amboin. 6: 140, t. 62, 63 1750.
Alpinia molucana Gagnep., Bull. Soc. Bot. France 48: XC 1901. Type: Indonesia. Moluccas: Pulau Rawak [Lawak], C. Gaudichaud 101 (lectotype: designated by Smith, Edin J. Bot. 47(1): 51 1990, P* [P00686668]).
Alpinia colossea K.Schum., Bot. Jahrb. Syst. 27: 289 1899. Type: Indonesia. West Papua, Soron [Sorong], O. Beccari 192 (lectotype: designated by Cecchi et al. Plant Biosyst. 156(3): 782 2022. FI! [FI008301]).

#### ﻿ Hellwigia odontonema

Taxon classificationPlantaeZingiberalesZingiberaceae

(K.Schum.) Senjaya & A.D.Poulsen comb. nov.

BD61B532-EE9E-596C-BE0E-3CACCBBB4EA9

urn:lsid:ipni.org:names:77367810-1

Alpinia odontonema K.Schum., Nachtr. Fl. Schutzgeb. Südsee 65 1905 (Basionym). Type: Papua New Guinea. Bismarck Mountains, F.R.R. Schlechter 1395 (lectotype: designated by Smith, 36(2): 277 1978, B n.v., assumed lost in the Second World War).
Alpinia brevituba Valeton, Nova Guinea 8: 953 1913. Type: Indonesia. Papua: Hellwig Mountain, L.S.A.M. von Römer 1109 (lectotype: designated here, BO! [BO-0081536]).

##### Note.

As Valeton (1916) and Smith (1990a) did not mention where the type was deposited, we designate the BO material as the lectotype.

#### ﻿ Hellwigia oligantha

Taxon classificationPlantaeZingiberalesZingiberaceae

(Valeton) Senjaya & A.D.Poulsen comb. nov.

D04A8D03-7B49-5BC5-8259-C7458484622E

urn:lsid:ipni.org:names:77367811-1

Alpinia oligantha Valeton, Nova Guinea 8: 957 (1913) (Basionym). Type: Indonesia: Papua, Versteeg 1656 (lectotype: designated here, BO! [BO-0034516]).

##### Note.

Smith (1990a) mentioned the type but not the herbarium. Therefore, we designate the BO material as the lectotype.

#### ﻿ Hellwigia orientalis

Taxon classificationPlantaeZingiberalesZingiberaceae

(Docot & Banag) Senjaya & A.D.Poulsen comb. nov.

4E7E97EC-FF25-5114-B1EC-3AE5AD317100

urn:lsid:ipni.org:names:77367812-1

Alpinia orientalis Docot & Banag, Kew Bulletin 77: 545 2022 (Basionym). Type: Philippines. Mindanao: Davao Oriental, San Isidro, Barangay La Union, Sitio Tumalite, Mount Hamiguitan, R.V. A. Docot, C. I. Banag & D. N. Tandang 0088 (holotype: PNH* [Sheet No. 258681], isotypes: E, FEUH, NY, USTH).

#### ﻿ Hellwigia papuana

Taxon classificationPlantaeZingiberalesZingiberaceae

(Scheff.) Senjaya & A.D.Poulsen comb. nov.

9D04C1B8-B704-5651-828B-582E653D1658

urn:lsid:ipni.org:names:77367813-1

Alpinia papuana Scheff., Ann. Jard. Bot. Buitenzorg 1: 56 1876 (Basionym). Alpinia gigantea var. papuana (Scheff.) Valeton in Nova Guinea 8: 943 1913. Type: Indonesia. West Papua: Sorong, J.E. Teijsmann s.n. (Sept 1871) (lectotype: designated by Smith, Notes Roy. Bot. Gard. Edinburgh 34(2): 161 1975, BO n.v.).
Alpinia eustales K. Schum., Bot. Jahrb. Syst. 27: 288 1899. Type: Indonesia. West Papua, O. Beccari 251 (lectotype: designated by Cecchi et al. Plant Biosyst. 156(3): 783 2022. FI! [FI008307])

##### Notes.

As no collection of *H. papuana* is available, *A. eustales*, placed in synonymy by Smith (1975), remains tentative. We disagree with Schumann (1904) in placing *A. collosea* as a synonym of *A. papuana*; instead, here we place it in synonymy with *H. nutans*.

#### ﻿ Hellwigia parksii

Taxon classificationPlantaeZingiberalesZingiberaceae

(Gillespie) Senjaya & A.D.Poulsen comb. nov.

79F85222-AF9B-555B-AEE9-1BBCE55C7E77

urn:lsid:ipni.org:names:77367814-1

Languas parksii Gillespie, Bishop Mus. Bull. 91: 4 f.4 1932 (Basionym). Alpinia parksii (Gillespie) A.C. Smith, Sargentia 1: 7 1942. Type: Fiji. Viti Levu: Namosi Province, Parks 20379 (holotype: BISH* [BISH1005399]).

#### ﻿ Hellwigia platylopha

Taxon classificationPlantaeZingiberalesZingiberaceae

(Ridl.) Senjaya & A.D.Poulsen comb. nov.

96A9BE83-02DE-5F7F-B923-2E9CD9B42A1A

urn:lsid:ipni.org:names:77367815-1

Eriolopha platylopha Ridl., Trans. Linn. Soc. London, Bot. 9: 220 1916 (Basionym). Alpinia platylopha (Ridl.) Loes., Nat. Pflanzenfam. ed. 2, 15a: 622 1930. Type: Indonesia. Papua: Mount Carstensz, Camp VIc, C.B. Kloss s.n. (17 Feb 1913) (lectotype: second step, designated here BM* [BM000617297]).

##### Note.

Smith (1975) mentioned the type at BM but did not specify the locality and date.

#### ﻿ Hellwigia porphyrea

Taxon classificationPlantaeZingiberalesZingiberaceae

(R.M.Sm.) Senjaya & A.D.Poulsen comb. nov.

991DC98E-8FDF-5FC7-BE3C-40D7490AB3B6

urn:lsid:ipni.org:names:77367816-1

Alpinia porphyrea R.M. Sm., Notes Roy. Bot. Gard. Edinburgh 36: 287 1978 (Basionym). Type: Indonesia. West Papua: South Sorong, Teminabuan, C. Kalkman 6262 (holotype: L! [L 1468301], isotype E! [E00149554]).

#### ﻿ Hellwigia porphyrocarpa

Taxon classificationPlantaeZingiberalesZingiberaceae

(Ridl.) Senjaya & A.D.Poulsen comb. nov.

43568BC3-747C-5E2F-8AAC-422605D650E9

urn:lsid:ipni.org:names:77367843-1

Alpinia porphyrocarpa Ridl. Trans. Linn. Soc. London, Bot. 9: 213 1916 (Basionym). Type: Indonesia. Papua: Utakwa River to Mt. Carstensz, Canoe Camp, C.B. Kloss s.n. (June 1913) (lectotype: designated by Turner Asian J. Trop. Biol. 4: 13 2000. (lectotype: K* [K000292434]; isolectotype: BM n.v.).

##### Notes.

Smith (1975, 1990a) and Turner (2000) mentioned the type being at BM and K, but we are unable to locate the specimen at the former herbarium.

#### ﻿ Hellwigia pulchra

Taxon classificationPlantaeZingiberalesZingiberaceae

Warb., Bot. Jahrb. Syst. 13: 279 1891.

DA9C828D-B780-5A31-997F-D4D618B579E4

Alpinia pulchra (Warb.) K.Schum. Pflanzenr. IV, 46: 348. 1904. Type: Papua New Guinea, Sattelberg, Mar–Apr 1889, O. Warburg s.n. (lectotype: designated here, Warburg 21059 (BM* [BM014605304]); (isolectotype: B n.v., assumed lost during the Second World War).
Alpinia stapfiana K. Schum., Pflanzenr. IV, 46: 347 1904. Type: Solomon Islands. Shortland Island, Guppy 103 (lectotype: designated by Smith, Notes Roy. Bot. Gard. Edinburgh 36(2): 289 1978, K*[K000928011]; isolectotype: E! [E00149583]).
Alpinia rechingeri Gagnep., Bull. Soc. Bot. France 55: 432 1908. Type: Solomon Islands. Shortland Island, K. & L. Rechinger 3859 (lectotype: designated by Smith, Notes Roy. Bot. Gard. Edin. 36(2): 289 1978, P n.v.; isolectotype: E! [E00149561]).

#### ﻿ Hellwigia regia

Taxon classificationPlantaeZingiberalesZingiberaceae

(K.Heyne ex R.M.Sm.) Senjaya & A.D.Poulsen comb. nov.

304930CF-25F5-5327-908A-12AFB204E63C

urn:lsid:ipni.org:names:77367817-1

Alpinia regia K.Heyne ex R.M.Sm., Notes Roy. Bot. Gard. Edinburgh 35: 203 1977 (Basionym). Type: Indonesia. Moluccas: Ternate, V.M.A. Beguin 1234 (holotype: L! [L 0041080, L 0599625]; isotypes: BO! [Sheet Nos. BO-0080602, 0080603, 0080605], E! [E00149617]).

#### ﻿ Hellwigia rigida

Taxon classificationPlantaeZingiberalesZingiberaceae

(Ridl.) Senjaya & A.D.Poulsen comb. nov.

22269DD9-2803-5C90-9BF1-6FC318BF8663

urn:lsid:ipni.org:names:77367818-1

Alpinia rigida Ridl., Trans. Linn. Soc. London, Bot. 9: 214 1916 (Basionym). Eriolopha rigida Ridl., Trans. Linn. Soc. London, Bot. 9: 217 1916. Type: Indonesia. Papua, Utakwa River to Mt. Carstensz, Camp VIa, C.B. Kloss s.n. (17 Jan 1913) (lectotype: designated by Smith, Notes Roy. Bot. Gard. Edin. 34(2): 175 1975, BM! [BM000617268].

#### ﻿ Hellwigia rosacea

Taxon classificationPlantaeZingiberalesZingiberaceae

(Valeton) Senjaya & A.D.Poulsen comb. nov.

21E0AF82-3AAE-58B8-B58D-0E94A7A37576

urn:lsid:ipni.org:names:77367819-1

Alpinia rosacea Valeton, Nova Guinea 8: 945 1913 (Basionym). Types: Indonesia. Papua, G.M. Versteeg 1655 (lectotype: designated here, L! [L 0360117]; isolectotypes: BO! [Sheet Nos. BO-0081771, 773], E! [E00149699], K* [K000292431–432]),1227 (syntype: BO! [Sheet No. BO-0081769], K n.v.), B. Branderhorst 333 (syntype: BO! [Sheet No. BO-0081766]).

##### Note.

Smith (1990a) listed the three syntypes, of which we select here the superior one as the lectotype.

#### ﻿ Hellwigia samoensis

Taxon classificationPlantaeZingiberalesZingiberaceae

(Reinecke) Senjaya & A.D.Poulsen comb. nov.

6BE74CBF-D43E-5171-9C93-4E9719997235

urn:lsid:ipni.org:names:77367820-1

Alpinia samoensis Reinecke, Bot. Jahrb. Syst. 25: 597 f.10 1898 (Basionym). Type: Samoa. Reinecke 301 (lectotype: designated here, E! [E00149782]).
Alpinia dyeri K. Schum., Pflanzenr. IV, 46: 349 1904. Type: Samoa. S.J. Whitmee 276 (lectotype: designated here, K* [K000928024]).

##### Note.

Smith (1990a) did not specify the herbarium of the type, which is the reason for selecting the E type of *H. samoensis* here.

#### ﻿ Hellwigia sandsii

Taxon classificationPlantaeZingiberalesZingiberaceae

(R.M.Sm.) Senjaya & A.D.Poulsen comb. nov.

723D5852-BDE0-5E7F-8509-E894A794E638

urn:lsid:ipni.org:names:77367821-1

Alpinia sandsii R.M.Sm., Edinburgh J. Bot. 48: 347 1991 (Basionym). Type: Indonesia. South Sulawesi: Enrekang, Latimojong Mountains, M.J.S. Sands 459 (holotype: K! [K000292390-391], isotype: E! [E00149619–621]).

#### ﻿ Hellwigia schultzei

Taxon classificationPlantaeZingiberalesZingiberaceae

(Lauterb. ex Valeton) Senjaya & A.D.Poulsen comb. nov.

EDA86AFC-E0AD-53F1-B0B5-3D5992649FDB

urn:lsid:ipni.org:names:77367822-1

Alpinia schultzei Lauterb ex Valeton Bot. Jahrb. Syst. 52: 69 1914 (Basionym). Type: Papua New Guinea. Sepik River, Bivak 48, Schultze 270 (n.v.).

##### Note.

Smith was also unable to locate the type.

#### ﻿ Hellwigia sericea

Taxon classificationPlantaeZingiberalesZingiberaceae

(Ridl.) Senjaya & A.D.Poulsen comb. nov.

F72F9869-D700-5874-8D0D-76A1D193687C

urn:lsid:ipni.org:names:77367823-1

Eriolopha sericea Ridl., Trans. Linn. Soc. London, Bot. 9: 221 1916 (Basionym). Alpinia bodenii R.M.Sm., Edinburgh J. Bot. 47: 64 1990. Type: Indonesia. Papua: Mt. Carstensz, Camp VIa, C.B. Kloss s.n. (17 Jan 1913) (lectotype: designated by Turner, Asian J. Trop. Biol. 4: 22 2000. BM* [BM000617292]; isolectotype: K! [K000292449]).

#### ﻿ Hellwigia sericiflora

Taxon classificationPlantaeZingiberalesZingiberaceae

(K.Schum.) Senjaya & A.D.Poulsen comb. nov.

F053C7D7-C796-55DE-92B2-606A041710D3

urn:lsid:ipni.org:names:77367824-1

Alpinia sericiflora K.Schum., Bot. Jahrb. Syst. 27: 294 1899 (Basionym). Type: Indonesia. Aru Islands, O. Beccari 7 (lectotype: designated by Cecchi et al. Plant Biosyst. 156(3): 785 2022. FI! [FI008319]).

#### ﻿ Hellwigia singuliflora

Taxon classificationPlantaeZingiberalesZingiberaceae

(R.M.Sm.) Senjaya & A.D.Poulsen comb. nov.

8744EA1D-010E-5F93-A227-8ED33DCC764F

urn:lsid:ipni.org:names:77367825-1

Alpinia singuliflora R.M.Sm., Notes Roy. Bot. Gard. Edinburgh 36: 279 1978 (Basionym). Type: Papua New Guinea. Southern Highlands, J.R. Croft LAE 60850 (holotype: E! [E00149566]; isotype: LAE n.v.).

#### ﻿ Hellwigia salomonensis

Taxon classificationPlantaeZingiberalesZingiberaceae

(B.L.Burtt & R.M.Sm.) Senjaya & A.D.Poulsen comb. nov.

44207723-1DC7-592A-BD51-D163C2AF3035

urn:lsid:ipni.org:names:77367831-1

Alpinia salomonensis B.L.Burtt & R.M.Sm., Notes Roy. Bot. Gard. Edinburgh 32: 41 1972 (Basionym). Type: Solomon Islands. Guadalcanal: Popomanasiu, Corner 143 (holotype K* [K000928004–005]; isotype E! [E00149648–650]).

#### ﻿ Hellwigia stenobracteolata

Taxon classificationPlantaeZingiberalesZingiberaceae

(R.M.Sm.) Senjaya & A.D.Poulsen comb. nov.

CAB13064-5EC4-513E-AC24-CFE17B351FAC

urn:lsid:ipni.org:names:77367826-1

Alpinia stenobracteolata R.M.Sm., Notes Roy. Bot. Gard. Edinburgh 36: 291 1978 (Basionym). Type: Papua New Guinea. Chimbu, A.N. Millar NGF 37800 (holotype: LAE! [Sheet No. 106235]; isotypes: A* [00030628], E! [E00149567, E00275012], L! [L 04428422–423]).
Alpinia squarrosa Gilli, Ann. Naturhist. Mus. Wien, 84B: 44 1983. Type: Papua New Guinea. Western Highlands, A. Gilli 543 (holotype: W* [W 1979-0016648]).

#### ﻿ Hellwigia strobilacea

Taxon classificationPlantaeZingiberalesZingiberaceae

(K.Schum.) Senjaya & A.D.Poulsen comb. nov.

C7A89C84-C9AE-5769-829B-AB5A7A8F4EA6

urn:lsid:ipni.org:names:77367827-1

Alpinia strobilacea K.Schum., Bot. Jahrb. Syst. 27: 295 1899 (Basionym). Type: Indonesia. West Papua, Andai, O. Beccari 522 (lectotype: designated by Cecchi et al. Plant Biosyst. 156(3): 785 2022. FI! [FI008320]).
Eriolopha seticalyx Ridl., Trans. Linn. Soc. London, Bot. 9: 221 1916. Alpinia seticalyx (Ridl.) Loes., Nat. Pflanzenfam. ed. 2, 15a: 620 1930. Type: Indonesia. Papua: Utakwa River to Mt. Carstenz, Canoe Camp, C.B. Kloss s.n. (Nov 1912) (lectotype: designated by Smith, Edin. J. Bot. 47(1): 58 1990, K! [K000292430]; isolectotype: BM! [BM000617296]).

#### ﻿ Hellwigia subverticillata

Taxon classificationPlantaeZingiberalesZingiberaceae

(Valeton) Senjaya & A.D.Poulsen comb. nov.

9A2343FA-8B9D-5983-A8CA-4F757D489AF9

urn:lsid:ipni.org:names:77367828-1

Alpinia subverticillata Valeton, Nova Guinea 8: 950 1913 (Basionym). Types: Indonesia. Papua, G.M. Versteeg 1606 (lectotype: designated here, K! [K000292428–429]; isolectotype: BO! [Sheet Nos. BO-0081102, 104]), G.M. Versteeg 1416 (syntype: BO! [Sheet No. BO-0081103], L n.v.).

##### Note.

We designate the K collection as the lectotype because of its better condition.

#### ﻿ Hellwigia superba

Taxon classificationPlantaeZingiberalesZingiberaceae

(Ridl.) Senjaya & A.D.Poulsen comb. nov.

E10667F4-55DC-5EC4-9B4C-7E0D4A86496E

urn:lsid:ipni.org:names:77367829-1

Guillainia superba Ridl., Trans. Linn. Soc. London, Bot. 9: 216 1916 (Basionym). Alpinia superba (Ridl.), Nat. Pflanzenfam. ed. 2, 15a: 622 1930. Type: Indonesia. Papua: Utakwa River to Mt. Carstensz, Camp VIb, C.B. Kloss s.n. (24 Jan 1913) (lectotype: designated by Turner, Asian J. Trop. Biol. 4: 32 2000. BM! [BM000617554]; isolectotype: K! [K000292427]).

#### ﻿ Hellwigia tristachya

Taxon classificationPlantaeZingiberalesZingiberaceae

(Ridl.) Senjaya & A.D.Poulsen comb. nov.

789C8CC2-0F87-5268-ACEF-60DAD0965477

urn:lsid:ipni.org:names:77367830-1

Eriolopha tristachya Ridl., Trans. Linn. Soc. London, Bot. 9: 218 1916 (Basionym). Alpinia tristachya (Ridl.) Loes., Nat. Pflanzenfam. ed. 2, 15a: 622 1930. Type: Indonesia. Papua, Camp VIa, C.B. Kloss s.n. (17 Jan 1913) (lectotype: designated here, BM! [BM000617346]; isolectotypes: E! [00149702], K! [K000292426], SING).

##### Note.

We select the duplicate at BM as the lectotype because it has the best quality.

#### ﻿ Hellwigia unilateralis

Taxon classificationPlantaeZingiberalesZingiberaceae

(B.L.Burtt & R.M.Sm.) Senjaya & A.D.Poulsen comb. nov.

CD038327-3B2C-5D5D-A4F8-E56FFED0649B

urn:lsid:ipni.org:names:77367832-1

Alpinia unilateralis B.L.Burtt & R.M.Sm., Notes Roy. Bot. Gard. Edinburgh 32: 37 1972 (Basionym). Type: Solomon Islands. Guadalcanal, Corner 106 (holotype: K! [K000928016]; isotypes: E! [E00149647], L! [L 0041082], WAG* [WAG 1202058]).

#### ﻿ Hellwigia velutina

Taxon classificationPlantaeZingiberalesZingiberaceae

(P.Royen) Senjaya & A.D.Poulsen comb. nov.

F65CCF04-59E0-5DF6-90CE-88C195487DA6

urn:lsid:ipni.org:names:77367834-1

Kolowratia velutina P.Royen, The Alpine Flora of New Guinea 2: 887 1979 (Basionym). Alpinia velveta R.M.Sm., Edinburgh J. Bot. 47: 63 1990. Type: Papua New Guinea. Eastern Highlands, L.J. Brass 30553 (holotype: L! [L 0041085]; isotypes: A* [00030667], E! [E00149770], LAE! [Sheet No. 36026]).

#### ﻿ Hellwigia vitiensis

Taxon classificationPlantaeZingiberalesZingiberaceae

(Seem.) Senjaya & A.D.Poulsen comb. nov.

6228A3A2-D82A-5747-BC66-3B423E566C50

urn:lsid:ipni.org:names:77367835-1

Alpinia vitiensis Seem., Fl. Vit.: 290 1868 (Basionym). Type: Fiji. Taviuni, Seeman 621 (lectotype: designated by Smith, Edin. J. Bot. 47(1): 61 1990, K! [K000928018]; isolectotypes: A* [00030644], BM* [BM000990753]).

#### ﻿ Hellwigia vulcanica

Taxon classificationPlantaeZingiberalesZingiberaceae

(Elmer) Senjaya & A.D.Poulsen comb. nov.

ED76AF38-25CD-5649-8C1B-58BBD3805BA8

urn:lsid:ipni.org:names:77367836-1

Alpinia vulcanica Elmer, Leafl. Philipp. Bot. 8: 2971 1919 (Basionym). Languas vulcanica (Elmer) Merr., Enum. Philipp. fl. pl. 1: 234 1923. Type: Philippines. Sorsogon: Irosin, Mt. Bulusan, A.D.E. Elmer 16168 (lectotype: designated by Docot et al. Kew Bull. 77: 549 2022. NY* [00320197]; isolectotypes: A* [00030637], BO! [Sheet No. BO-0081092], F, GH, MO).

#### ﻿ Hellwigia werneri

Taxon classificationPlantaeZingiberalesZingiberaceae

(Lauterb. ex Valeton) Senjaya & A.D.Poulsen comb. nov.

C5E564EC-F6F6-5C51-A7B4-62F23C5EB7C5

urn:lsid:ipni.org:names:77367837-1

Alpinia werneri Lauterb. ex Valeton, Bot. Jahrb. Syst. 52: 69 1914 (Basionym). Type: Papua New Guinea, Kami Mountains, F.R.R. Schlechter 17654 (lectotype: designated here, K* [K000292424]; isolectotype: E! [E00149574, flower only, E00275011, photocopy]).

##### Note.

As the sheet at K includes leaves and an inflorescence, it is chosen as the lectotype.

#### ﻿ Hellwigia womersleyi

Taxon classificationPlantaeZingiberalesZingiberaceae

(R.M.Sm.) Senjaya & A.D.Poulsen comb. nov.

92632AB7-29EC-5C7A-8F02-626C4138E4B1

urn:lsid:ipni.org:names:77367838-1

Alpinia womersleyi R.M.Sm., Notes Roy. Bot. Gard. Edinburgh 36: 285 1978 (Basionym). Type: Papua New Guinea, Western Highlands, J.S. Womersley, J. Vandenburg & M. Galore NGF 37268 (holotype: LAE! [Sheet No. 105424]; isotypes: E! [E00149575, E00275010], L! [L 0428398]).

## Supplementary Material

XML Treatment for Hellwigia

XML Treatment for Hellwigia acuminata

XML Treatment for Hellwigia aenea

XML Treatment for Hellwigia albipurpurea

XML Treatment for Hellwigia arfakensis

XML Treatment for Hellwigia athroantha

XML Treatment for Hellwigia biakensis

XML Treatment for Hellwigia boia

XML Treatment for Hellwigia calycodes

XML Treatment for Hellwigia carinata

XML Treatment for Hellwigia carolinensis

XML Treatment for Hellwigia celebica

XML Treatment for Hellwigia chaunocolea

XML Treatment for Hellwigia coeruleoviridis

XML Treatment for Hellwigia conferta

XML Treatment for Hellwigia conglomerata

XML Treatment for Hellwigia cylindrocephala

XML Treatment for Hellwigia dasystachys

XML Treatment for Hellwigia dekockii

XML Treatment for Hellwigia densiflora

XML Treatment for Hellwigia divaricata

XML Treatment for Hellwigia domatifera

XML Treatment for Hellwigia eremochlamys

XML Treatment for Hellwigia euastra

XML Treatment for Hellwigia flagellaris

XML Treatment for Hellwigia gigantea

XML Treatment for Hellwigia glacicaerulea

XML Treatment for Hellwigia gracillima

XML Treatment for Hellwigia hagena

XML Treatment for Hellwigia himantoglossa

XML Treatment for Hellwigia horneana

XML Treatment for Hellwigia inaequalis

XML Treatment for Hellwigia janowskii

XML Treatment for Hellwigia juliformis

XML Treatment for Hellwigia kiungensis

XML Treatment for Hellwigia klossii

XML Treatment for Hellwigia laxisecunda

XML Treatment for Hellwigia leptostachya

XML Treatment for Hellwigia macrocarpa

XML Treatment for Hellwigia manostachys

XML Treatment for Hellwigia maxii

XML Treatment for Hellwigia monopleura

XML Treatment for Hellwigia multispica

XML Treatment for Hellwigia musifolia

XML Treatment for Hellwigia nidus-vespae

XML Treatment for Hellwigia novae-hiberniae

XML Treatment for Hellwigia nutans

XML Treatment for Hellwigia odontonema

XML Treatment for Hellwigia oligantha

XML Treatment for Hellwigia orientalis

XML Treatment for Hellwigia papuana

XML Treatment for Hellwigia parksii

XML Treatment for Hellwigia platylopha

XML Treatment for Hellwigia porphyrea

XML Treatment for Hellwigia porphyrocarpa

XML Treatment for Hellwigia pulchra

XML Treatment for Hellwigia regia

XML Treatment for Hellwigia rigida

XML Treatment for Hellwigia rosacea

XML Treatment for Hellwigia samoensis

XML Treatment for Hellwigia sandsii

XML Treatment for Hellwigia schultzei

XML Treatment for Hellwigia sericea

XML Treatment for Hellwigia sericiflora

XML Treatment for Hellwigia singuliflora

XML Treatment for Hellwigia salomonensis

XML Treatment for Hellwigia stenobracteolata

XML Treatment for Hellwigia strobilacea

XML Treatment for Hellwigia subverticillata

XML Treatment for Hellwigia superba

XML Treatment for Hellwigia tristachya

XML Treatment for Hellwigia unilateralis

XML Treatment for Hellwigia velutina

XML Treatment for Hellwigia vitiensis

XML Treatment for Hellwigia vulcanica

XML Treatment for Hellwigia werneri

XML Treatment for Hellwigia womersleyi

## ﻿Additional information

### Use of AI

No use of AI was reported.

### Author ORCIDs

Seni Kurnia Senjaya https://orcid.org/0000-0003-4952-1352

Axel Dalberg Poulsen https://orcid.org/0000-0002-7651-6439

Marlina Ardiyani https://orcid.org/0000-0003-2453-3066

Quentin C.B. Cronk https://orcid.org/0000-0002-4027-7368

### Data availability

The data supporting this study’s findings are openly available in GenBank (https://www.ncbi.nlm.nih.gov/genbank/) and Dryad (https://doi.org/10.5061/dryad.g1jwstr2t), with the sequences generated in this study accessible in GenBank under accession numbers PV158122–PV158191.

## Supplementary materials

Supplementary material 1 Occurrence data for voucher specimens Data type csv Explanation note The occurrence data of specimens used to generate the distribution map of the *Alpinia* "Carolinensis" clade include geographic coordinates, collection localities, and voucher specimen details. https://binary.pensoft.net/file/1397253This dataset is made available under the Open Database License (http://opendatacommons.org/licenses/odbl/1.0/). The Open Database License (ODbL) is a license agreement intended to allow users to freely share, modify, and use this Dataset while maintaining this same freedom for others, provided that the original source and author(s) are credited.Seni Kurnia Senjaya, Axel Dalberg Poulsen, Marlina Ardiyani, Quentin C.B. Cronk

Supplementary material 2 DNA sequence alignment matrix Data type fasta Explanation note The DNA alignment matrix consists of sequence data from all analyzed specimens. https://binary.pensoft.net/file/1397254This dataset is made available under the Open Database License (http://opendatacommons.org/licenses/odbl/1.0/). The Open Database License (ODbL) is a license agreement intended to allow users to freely share, modify, and use this Dataset while maintaining this same freedom for others, provided that the original source and author(s) are credited.Seni Kurnia Senjaya, Axel Dalberg Poulsen, Marlina Ardiyani, Quentin C.B. Cronk

Supplementary material 3 Phylogenetic tree of Alpinioideae inferred from maximum likelihood (ML) and Bayesian analyses Data type svg Explanation note Phylograms of the Alpinioideae subfamily inferred from the nuclear ribosomal Internal Transcribed Spacer (ITS) dataset. Left: Maximum Likelihood (ML) tree. Right: Bayesian consensus tree. Red lines indicate strong branch support, with bootstrap values (≥75%) in the ML tree and posterior probabilities (≥0.75) in the Bayesian tree. Bootstrap values between 50% and 74%, as well as posterior probabilities between 0.5 and 0.74, are annotated above branches or indicated with arrows. https://binary.pensoft.net/file/1397255This dataset is made available under the Open Database License (http://opendatacommons.org/licenses/odbl/1.0/). The Open Database License (ODbL) is a license agreement intended to allow users to freely share, modify, and use this Dataset while maintaining this same freedom for others, provided that the original source and author(s) are credited.Seni Kurnia Senjaya, Axel Dalberg Poulsen, Marlina Ardiyani, Quentin C.B. Cronk

Supplementary material 4 Data table of voucher specimens and their corresponding GenBank accession numbers used in this study Data type xlsx Explanation note The table includes a comprehensive list of voucher specimens and their corresponding GenBank accession numbers used in this study. The table also provides the relevant references for sequences obtained from other publications, ensuring proper attribution and traceability of previously published data. https://binary.pensoft.net/file/1397256This dataset is made available under the Open Database License (http://opendatacommons.org/licenses/odbl/1.0/). The Open Database License (ODbL) is a license agreement intended to allow users to freely share, modify, and use this Dataset while maintaining this same freedom for others, provided that the original source and author(s) are credited.Seni Kurnia Senjaya, Axel Dalberg Poulsen, Marlina Ardiyani, Quentin C.B. Cronk
